# The Lineage Contribution and Role of *Gbx2* in Spinal Cord Development

**DOI:** 10.1371/journal.pone.0020940

**Published:** 2011-06-16

**Authors:** Brian Luu, Debra Ellisor, Mark Zervas

**Affiliations:** Division of Biology and Medicine, Department of Molecular Biology, Cell Biology and Biochemistry, Brown University, Providence, Rhode Island, United States of America; Seattle Children's Research Institute, United States of America

## Abstract

**Background:**

Forging a relationship between progenitors with dynamically changing gene expression and their terminal fate is instructive for understanding the logic of how cell-type diversity is established. The mouse spinal cord is an ideal system to study these mechanisms in the context of developmental genetics and nervous system development. Here we focus on the *Gastrulation homeobox 2* (*Gbx2*) transcription factor, which has not been explored in spinal cord development.

**Methodology/Principal Findings:**

We determined the molecular identity of *Gbx2*-expressing spinal cord progenitors. We also utilized genetic inducible fate mapping to mark the *Gbx2* lineage at different embryonic stages *in vivo* in mouse. Collectively, we uncover cell behaviors, cytoarchitectonic organization, and the terminal cell fate of the *Gbx2* lineage. Notably, both ventral motor neurons and interneurons are derived from the *Gbx2* lineage, but only during a short developmental period. Short-term fate mapping during mouse spinal cord development shows that *Gbx2* expression is transient and is extinguished ventrally in a rostral to caudal gradient. Concomitantly, a permanent lineage restriction boundary ensures that spinal cord neurons derived from the *Gbx2* lineage are confined to a dorsal compartment that is maintained in the adult and that this lineage generates inhibitory interneurons of the spinal cord. Using lineage tracing and molecular markers to follow *Gbx2*-mutant cells, we show that the loss of *Gbx2* globally affects spinal cord patterning including the organization of interneuron progenitors. Finally, long-term lineage analysis reveals that the presence and timing of *Gbx2* expression in interneuron progenitors results in the differential contribution to subtypes of terminally differentiated interneurons in the adult spinal cord.

**Conclusions/Significance:**

We illustrate the complex cellular nature of *Gbx2* expression and lineage contribution to the mouse spinal cord. In a broader context, this study provides a direct link between spinal cord progenitors undergoing dynamic changes in molecular identity and terminal neuronal fate.

## Introduction

The spinal cord coordinates motor and sensory information and serves as a central conduit between the external environment and brain. The spinal cord has generated intense interest because of its relevance to disease and trauma, the extent and etiology of which is related to the diverse population of neurons underpinning spinal cord function. The spinal cord can be broadly partitioned into two anatomical and functionally distinct regions along the dorsal-ventral (D-V) axis. The dorsal spinal cord contains sensory neurons that process somatosensory modalities of touch, heat, and pain [1]. This information is relayed to ventral motor neurons as part of a reflex circuit and to brain centers including the brainstem, thalamus, and cerebellum as part of a higher order integrative circuit. In contrast, the ventral cord contains neurons that control proprioception and motor output [2], [3]. The cytoarchitecture of the spinal cord is organized into ten regions [4]: laminae I–VI in the dorsal gray matter horn, laminae VII–IX in ventral gray matter horn, and area X, which surrounds the central canal [5]. In addition to this spatial arrangement, diverse arrays of molecularly and physiologically distinct neuronal sub-populations with varying axonal projection patterns reside in each lamina [2], [3], [6], [7].

Because of the spinal cord's functional importance and clinical relevance a great amount of research has focused on how spinal cord neuron subtype diversity is established during embryonic development [6], [7]. Consequently, early spinal cord development has become an outstanding model system to study molecular signaling and the transcriptional regulation that controls nervous system patterning and cell fate specification during embryogenesis [8], [9]. During embryogenesis, graded Sonic Hedgehog (SHH) signaling from the floorplate patterns the ventral neural tube and establishes five molecularly distinct ventral neural progenitor domains [10]. In contrast, graded Wingless/Int (WNT) and bone morphogenic protein signaling from the roofplate pattern the dorsal neural tube to establish six dorsal progenitor domains [7], [11]. Furthermore, a precisely choreographed transcriptional code is required for spinal progenitors to acquire their early neuronal and positional identity [10], [12]. In addition, homeodomain or bHLH transcription factors exhibits cross-repressive effects that refine and maintain the D-V border between specified progenitors [8], [13]. Subsequent to specification, differentiating neurons express unique combinations of post-mitotic transcription factors to diversify regional cell fate, positioning, and axonal projection patterns [14]–[16]. This multi-step process occurs along the anterior-posterior (A-P) axis and is regulated in part by paraxial mesoderm [6].

Spinal cord progenitors undergo cell fate decisions that are intimately related to their invariant position in the adult spinal cord and that are determined by intricate molecular control mechanisms [17]. However, the spatial and temporal contribution of spinal cord progenitors based on their genetic history to the biochemically and functionally diverse neuronal subtypes in the developing and adult spinal cord is largely unresolved. We begin to address the link between progenitors, cell behaviors, and neuronal types directly with genetic lineage analysis in mouse. Specifically, we determine the cell fate of *Gbx2*-expressing progenitors marked at varying stages of embryonic spinal cord development. *Gbx2* is first expressed at the mid-streak stage during mouse embryogenesis [18], continues through mouse embryonic day (E)7.5 in all three germ layers [18], [19], and molecularly distinguishes the posterior domain of the developing embryo [19], [20]. *Gbx2* is expressed in the neural tube at E8.5 [20] and in the spinal cord from E9.5–E14.5 [19], [21], [22]. However, the molecular identity of *Gbx2*-expressing progenitors and their relationship to other spinal cord progenitors has not been determined. We hypothesized that the spatial expression and timing of *Gbx2* determines the cytoarchitectonic organization and cell fate of spinal cord neurons derived from this lineage. Functionally, *Gbx2* is temporally required for cerebellar development [23], for thalamic development [24], and for maintaining the midbrain/hindbrain boundary [20], [21]. Because of its functional requirement in other embryonic brain regions, we further hypothesized that *Gbx2* plays a functional role in spinal cord development.

To test these hypotheses, we utilized molecular analysis and a non-invasive Genetic Inducible Fate Mapping strategy (GIFM) [25]–[27] with *Gbx2^CreER-ires-eGFP^; R26R* mice [24]. This knock-in allelic configuration allowed us to mark progenitor cells of the *Gbx2* lineage with fine spatial and temporal control and to track them *in vivo*. The *Gbx2^CreER-ires-eGFP^* allele is advantageous because it can also be used to identify populations of neuronal progenitors dynamically expressing *Gbx2*. We show that the *Gbx2*-lineage marked at distinct early time points differentially contributes to spatially segregated sub-populations in the embryonic spinal cord including dorsal spinal cord interneuron precursors. By analyzing *Gbx2^CreER-ires-eGFP/CreER-ires-eGFP^* mutants and tracking mutant cells by GIFM we show that *Gbx2* loss of function causes aberrant spinal cord patterning at E10.5 and E12.5. Finally, long-term lineage analysis revealed that calbindin, GAD6, and Pax2 expressing subtypes of dorsal interneurons in the adult spinal cord were derived from progenitors that expressed *Gbx2* at specific time points during embryogenesis.

## Results

### Characterization and utility of *Gbx2^CreER-ires-eGFP^* mice in spinal cord development

**Figure 1:**
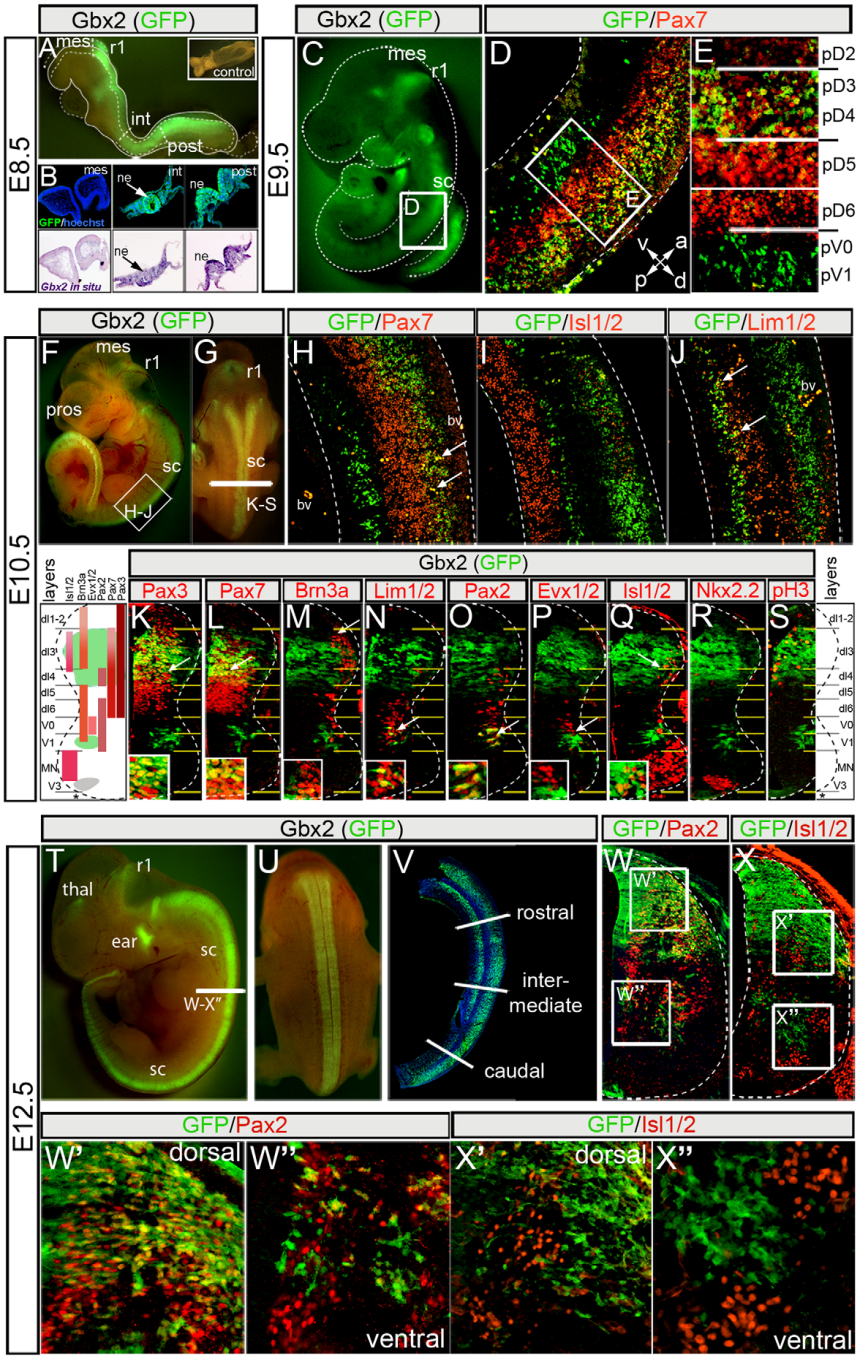
Dynamic expression of *Gbx2* in the developing spinal cord. *Gbx2*(GFP) expression detected in whole mount embryo (A). GFP immunolabeling (B, top row) and adjacent sections processed for *Gbx2 in situ* hybridization (B, bottom row) from E8.5 *Gbx2^CreER-ires-eGFP^* embryo; inset in “A” shows wildtype littermate. (C) *Gbx2*(GFP) expression in lateral view of an E9.5 embryo. (D–E) GFP and Pax7 immunolabeling on E9.5 *Gbx2^CreER-ires-eGFP/+^* sections. (F–G) Lateral (F) and (G) dorsal views of EGFP fluorescence in E10.5 *Gbx2^CreER-ires-eGFP/+^* embryo. (H–J) Antibody labeling of GFP and indicated markers on sagittal sections of E10.5 spinal cord; Note restricted ventral strip of *Gbx2*(GFP) expression (J, arrows). (K–S) Antibody labeling of GFP and indicated D-V markers on transverse hemi-sections of E10.5 spinal cord at the upper limb level. The insets show a high magnification view of the region indicated by the arrow. (T–U) EGFP fluorescence of E12.5 *Gbx2^CreER-ires-eGFP/+^* embryo showing lateral (T) and dorsal (U) view. (V) GFP antibody labeling on sagittal sections of E12.5 spinal cord. GFP/Pax2 (W–W″) and GFP/Isl1/2 (X–X″) immunolabeling on transverse E12.5 hemi-sections of spinal cord at the upper limb (rostral) level. Abbreviations: mesencephalon (mes), rhombomere 1 (r1), intermediate (int) and posterior (post) neural tube, neuroepithelium (ne), blood vessel (bv), prosencephalon (pros), thalamus (thal), spinal cord (sc).

To study *Gbx2* expression, lineage contribution, and function we utilized the mouse line, *Gbx2^CreER-ires-eGFP^* that was generated by targeting *CreER^T2^-ires-eGFP* to the 5′ untranslated region of Exon 1 in the *Gbx2* locus by homologous recombination [24]. In this configuration, the *eGFP* element allowed us to monitor *Gbx2* expression at the time of analysis by GFP whole mount fluorescence or by GFP antibody labeling of sections. Thus, we operationally defined *Gbx2*-expressing cells as being *Gbx2*(GFP)+. We validated that GFP accurately reflected endogenous *Gbx2* expression in the spinal cord by comparing anti-GFP antibody labeling to *in situ* hybridization with a labeled RNA probe specific to *Gbx2* on adjacent transverse sections from embryonic (E)8.5–E12.5 *Gbx2^CreER-ires-eGFP/+^* embryos (Figures 1 and S1). The *CreER^T2^* element [28], indicated as *CreER*, in the *Gbx2^CreER-ires-eGFP^* allele allowed us to perform GIFM [27], [29] (Figure S2). GIFM and tamoxifen administration indelibly marked the *Gbx2* lineage at distinct time points. With this approach and molecular marker analysis, we fate mapped and tracked the *Gbx2*-derived progeny, assessed their current state of *Gbx2* expression, and determined their molecular identity during development and in the adult. We also took advantage of this line to determine the functional requirement of *Gbx2* in spinal cord development by analyzing *Gbx2^CreER-ires-eGFP/CreER-ires-eGFP^* homozygous mutant embryos.

### The molecular identity of *Gbx2*-expressing spinal cord progenitors and differentiating neurons

*Gbx2*(GFP) in E8.5 *Gbx2^CreER-ires-eGFP/+^* embryos was expressed along the D-V axis and extended caudally from the border of the mesencephalon (mes) and rhombomere 1 (r1) posteriorly along the length of the neural tube (Figure 1A,B). These findings are consistent with *Gbx2* detected by *in situ* hybridization (Figure 1B) [21]. Whole mount fluorescence at E9.5 revealed that *Gbx2* continued to be expressed in r1 and was restricted to the caudal neural tube and presumptive spinal cord (Figure 1C). We used marker analysis and GFP immunocytochemistry to characterize the molecular identity of *Gbx2*(GFP)-expressing cells at E9.5. In the dorsal spinal cord, *Gbx2*(GFP) co-localized with the upper tier expression domain of Pax7, which is a transcription factor that defines dorsal interneuron progenitors and delineates the border between dorsal and ventral neural tube (Figure 1D) [8], [30], [31]. Closer inspection revealed that a combinatorial code of *Gbx2*(GFP)/marker expression defined five progenitor domains along the D-V axis at E9.5 (Figure 1E). The dorsal domains consisted of a (1) a *Gbx2* negative/weakly staining Pax7+ domain corresponding to pD1–pD2, (2) a domain of *Gbx2*(GFP)+/Pax7+ double positive cells corresponding to pD3–pD4, and (3) a *Gbx2*(GFP)−/Pax7+ domain corresponding to pD5–pD6 (Figure 1D,E). The ventral domains were comprised of (4) a cluster of *Gbx2*(GFP)+ cells located just ventral to the Pax7 border corresponding to pV0/V1 while (5) a more ventral domain was devoid of *Gbx2* and corresponded to pMN-pV3 (Figure 1D).

*Gbx2*(GFP) continued to be expressed in spinal cord, and to a lesser extent, in r1 at E10.5 (Figure 1F,G). Marker analysis at E10.5, when spinal progenitors acquire their neuronal subtype identity [7], [10], uncovered the spatial domains and molecular identity of *Gbx2*(GFP)-expressing cells (Figure 1H–S). Dorsal *Gbx2*(GFP)-expressing cells were localized to the upper tier of the Pax7 domain although there was less overlap than at E9.5 (Figure 1H versus 1D). *Gbx2(GFP)*-expressing cells did not overlap with ventral Isl1/2, dorsal Lim1/2-expressing cells, nor with the upper tier of the ventral Lim1/2 domain (Figure 1I–J). *Gbx2(GFP)*-expressing cells did overlap with the ventral tier of Lim1/2 (Figure 1J, arrows). We furthered our analysis by immunolabeling transverse sections with antibodies recognizing the following well-defined spinal cord markers (Figure 1, E10.5 schematic, reviewed in [1], [7], [8]): Pax3 (dl1–dl6), Pax7 (dl3–d6), Brn3a (lateral dl1–dl3, dl5-V1), Lim1/2 (dl6, V0–V1), Pax2 (dl4, dl6, V0–V1), Evx1/2 (V0), Isl1/2 (lateral dl3, MN), and Nkx2.2 (V3). We observed that *Gbx2*(GFP) was co-expressed with Pax3 (middle tier), Pax7 (upper tier), Lim1/2 (V1), Pax2 (V1), Isl1/2 (lateral dl3) (Figure 1K,L,N,O,Q). In contrast, *Gbx2*(GFP) did not co-localize with Brn3a, Evx1/2 (V0), ventral Isl1/2 (MN), or Nkx2.2 (V3) (Figure 1M,P,Q,R). *Gbx2*(GFP) expression was also not present in the roof plate or floor plate. Collectively, our marker analysis indicated that *Gbx2*(GFP) was expressed in molecularly and spatially distinct subsets of dorsal spinal cord neurons at E10.5. In contrast, *Gbx2*(GFP) expression was observed in V1 interneurons with few scattered neurons at the V0/V1 interface. *Gbx2*(GFP) expression was not in motor neurons or V3 interneurons. Analysis with the mitotic marker phosphorylated histone H3 (pH3) at E10.5 showed that dorsomedial *Gbx2*(GFP)-expressing cells were proliferating within the spinal cord ventricular zone (Figure 1S). In contrast, dorsolateral *Gbx2*(GFP)-expressing cells did not express pH3 and thus were non-mitotic cells that had migrated laterally from the ventricular zone. Ventral *Gbx2*(GFP)-expressing cells also did not co-express pH3 and therefore were non-mitotic (Figure 1S). In summary, dorsal *Gbx2*(GFP) expression at E10.5 delineated proliferating dI3–4 interneuron progenitors as they migrated laterally while *Gbx2*(GFP) ventrally primarily labels post-mitotic V1 interneurons.

**Figure 2:**
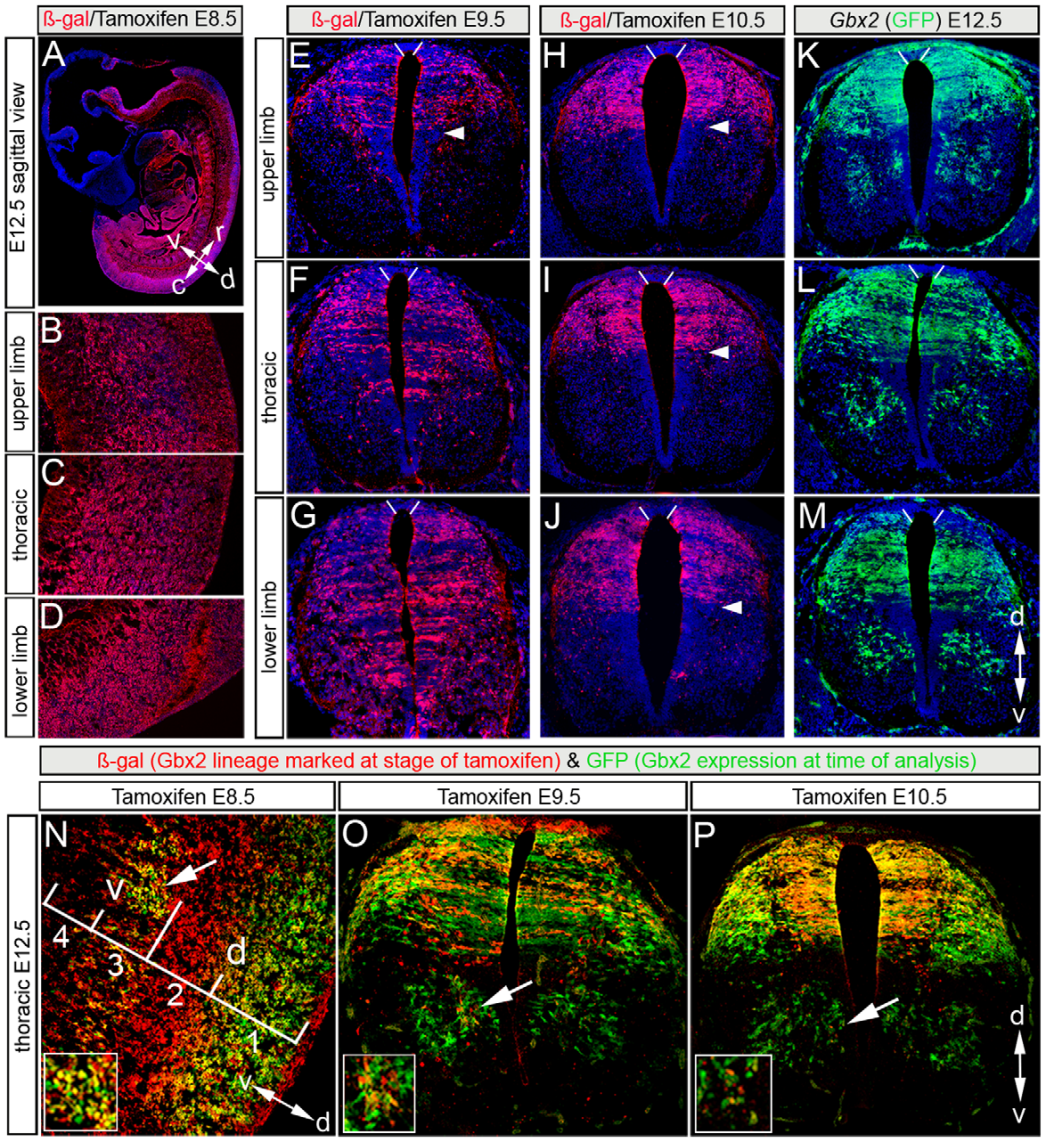
Spatial distribution of the *Gbx2* lineage in E12.5 spinal cord. (A–D) *Gbx2*-derived cells marked at E8.5 (ß-gal+, red) on sagittal sections of E12.5 spinal cord at the indicated levels. (E–G) Transverse sections of E12.5 spinal cord at the indicated levels showing ß-gal+ cells (red) that were marked at E9.5. (H–J) The *Gbx2* lineage (ß-gal+, red) marked at E10.5 was confined to the dorsal spinal cord at all axial levels at E12.5. (K–M) Transverse sections showing *Gbx2*(GFP)+ cells at indicated levels in E12.5 spinal cord. (N–P) Comparison of the *Gbx2* lineage (ß-gal+, red) marked at E8.5 (N), E9.5 (O), and E10.5 (P) versus *Gbx2* expression (GFP+, green) in E12.5 spinal cord. (N) Four D-V *Gbx2*-derived sub-populations can be classified by the presence or absence of *Gbx2*: zones 1 and 3 are *Gbx2*-derived cells that persisted in *Gbx2* expression while zones 2 and 4 have down-regulated *Gbx2*. (O) *Gbx2*-derived cells marked at E9.5 continued to express *Gbx2*(GFP) in dorsal spinal cord at E12.5 in contrast to few ventral cells. (P) The majority of *Gbx2*(GFP)-expressing cells marked at E10.5 were confined to a dorsal domain and continued to express *Gbx2*(GFP).

*Gbx2*(GFP) was strongly expressed along the A-P axis of the E12.5 spinal cord in a broad dorsal domain and in a ventral strip on sagittal sections (Figure 1T–V). The *Gbx2*(GFP) expression domain on transverse sections at E12.5 spanned the entire medial-lateral axis in the dorsal spinal cord (Figures 1W,X and 2K–M). In contrast, *Gbx2*(GFP) expression ventrally was limited to two well-delineated bi-lateral columns (Figures 1W,X and 2K–M). Notably, the *Gbx2*(GFP)+ domains exactly recapitulated the RNA *in situ* pattern of *Gbx2* expression at E12.5 (Figure S1). Compared to the expression pattern at E10.5, dorsal *Gbx2*(GFP) expression at E12.5 spanned a proportionally broader D-V domain and extended up to the dorsal-most point of spinal cord, excluding the roof plate (Figures 1K–R,1W–X). The *Gbx2*(GFP) cells dorsally expressed Pax2, a class-A transcription factor that defines late-born inhibitory interneurons at E12.5 [32] (Figure 1W,W′). *Gbx2*(GFP) expression co-localized more extensively with Pax2 dorsolaterally indicating that *Gbx2*(GFP) was also expressed in differentiating earlier-born inhibitory interneurons that have settled in the mantle and marginal zones (Figure 1W,W′). The most ventral *Gbx2*(GFP)-expressing cells only rarely co-expressed Pax2 (Figure 1W″). *Gbx2*(GFP) at E12.5 expression did not co-localize with dorsal Isl1/2 (Figure 1X,X′), which is a marker for dorsal excitatory interneurons [30]. To assess *Gbx2*(GFP) expression in motor neuron populations we analyzed ventral Isl1/2, which is a marker for all differentiating motor neurons in the ventral spinal cord [33]. Ventral-lateral *Gbx2*(GFP) expressing cells were nested within the Isl1/2+ medial and lateral motor columns (Figure 1X,X″), but did not did not co-localize with Isl1/2. Therefore, *Gbx2*(GFP) expression does not identify motor neuron sub-populations at E12.5, but *Gbx2*(GFP) expression at E12.5 defines early and late-born dorsal inhibitory interneuron sub-populations.

### Cell behaviors of the *Gbx2* lineage in spinal cord

To assess how *Gbx2* expression in progenitors during early to mid-embryogenesis related to the positional identity of Gbx2-derived cells as they differentiated into neurons, we marked and tracked the *Gbx2*-lineage using GIFM [26], [27] (Figure S2). We administered tamoxifen at E8.5, E9.5, or E10.5 and examined ß-gal immunolabeling in E12.5 spinal cords (Figure 2). Cells of the *Gbx2* lineage (ß-gal+) marked at E8.5 were distributed across the D-V axis along the full A-P extent of the spinal cord at E12.5 (Figure 2A–D). Therefore, *Gbx2*-expressing cells at the open neural tube stage (E8.5, Figure 1A,B) contributed substantially to the differentiating neurons of the spinal cord at E12.5 (Figures 2A–D and S3). In contrast, neurons of the *Gbx2* lineage marked by tamoxifen at E9.5 were differentially restricted based on their position along the A-P axis at E12.5 (Figures 2E–G and S4). At the upper limb level, *Gbx2*(GFP)-expressing cells marked at E9.5 were primarily concentrated in the dorsal spinal cord (Figures 2E and S4B). At the thoracic level, *Gbx2*-derived cells were primarily distributed in the dorsal cord and were present only sparsely in the ventral spinal cord (Figure 2F). At the lower limb level, *Gbx2*-derived cells marked at E9.5 contributed to the entire D-V extent of the spinal cord with the exception of the roof plate (Figures 2G and S4C). Therefore, *Gbx2^CreER^* plus tamoxifen at E8.5 marks ventral cells anteriorly (upper limb level) while marking at E9.5 does not (Figures 2B, S3, 2E and S4B). These findings indicate that between E8.5 and 9.5, the ventral *Gbx2*(GFP)-expressing progenitors begin to down regulate *Gbx2* rostrally. We then asked whether this wave of *Gbx2* down-regulation among ventral progenitors occurs in posterior spinal cord (lower limb level) over time. In sharp contrast to earlier marking, the *Gbx2* lineage marked at E10.5 was constrained by a tight boundary (Figures 2H–J, arrowhead) that confined marked neurons to a dorsal domain along the length of the spinal cord at E12.5 (Figures 2H–J and S5). Therefore, *Gbx2* expression is dynamic in progenitors and is temporally down-regulated in an A-P gradient, which progressively restricts the *Gbx2* lineage from contributing to the ventral spinal cord.

We also assessed the laminar distribution of *Gbx2* descendants marked at either E8.5 or E9.5 in adult spinal cord (Figure S6). The descendants of *Gbx2* progenitors marked at E8.5 were distributed across the full D-V and A-P extent of adult cord, extending from just posterior to the brain stem through the cauda equina (Figure S6A,B). In addition, X-gal labeling was seen in dorsal root ganglia (Figure S6B, arrows) and in cerebellar folia and in caudal hindbrain (Figure S6A,B). ß-gal immunolabeling on transverse sections showed that the *Gbx2* lineage marked at E8.5 spanned the entire dorsal-ventral and medial-lateral extent of gray matter at all A-P levels (Figure S6C–E). E8.5-marked descendants also populated the white matter at all three levels, although *Gbx2*-derived cells populated white matter less densely at the cervical versus lumbar level (Figure S6C–E). Thus, it appears that *Gbx2*-derived cells that contributed to the entire extent of the spinal cord at E12.5 were not substantially depleted during early embryogenesis, but rather persisted in their contribution to the spinal cord through the adult stage. Descendants from progenitors marked at E9.5 were concentrated in the dorsal region of the adult spinal cord, and consistent with the distribution at E12.5, did not contribute to rostral DRG (Figure S6F–J). X-gal activity was also detected in the cerebellum and in brainstem nuclei (Figures S6I,J). ß-gal immunofluorescence labeling on cross sections confirmed that E9.5-marked descendants were concentrated in the dorsal gray matter at the cervical level, with only a few cells residing in the dorsomedial white matter (Figure S6F). At the thoracic level, E9.5-marked descendants were in the dorsal horn, but were also scattered in more ventral cell populations (Figure S6G). At the lumbar level, E9.5-marked descendants spanned the full D-V extent of gray matter and were detected in the medial and lateral white matter (Figure S6H). These results suggest that once *Gbx2* was down-regulated in ventral progenitors beginning rostrally, the *Gbx2*-derived cells obeyed a lineage boundary that prevented cells from moving into ventral structures and together these mechanisms controlled the final cytoarchitectonic positioning of the *Gbx2* lineage in adult spinal cord.

### The regulation of *Gbx2* expression in the *Gbx2* lineage

We next determined whether *Gbx2*-derived cells continued to express *Gbx2* as they differentiated. The distribution of the *Gbx2* lineage (ß-gal+) (Figure 2A–J) was strikingly different from *Gbx2*(GFP) expression (Figure 2K–M) in differentiating neurons at E12.5. Double immunolabeling revealed that *Gbx2*(GFP)-expressing progenitors marked at E8.5 were partitioned into four sub-populations along the D-V axis in the maturing spinal cord based on persistent or transient *Gbx2*(GFP) expression (Figures 2N, S3A–G). The dorsal region consisted of a ß-Gal+/GFP+ cohort in the upper tier of the dorsal cord (Figure 2N, zone 1) and a ß-Gal+/GFP− population confined to the lower tier of the dorsal cord (Figure 2N, zone 2). A similar arrangement was observed in ventral spinal cord: an upper tier cohort of ß-Gal+/GFP+ cells and a lower tier of ß-Gal+/GFP− cells (Figure 2N, zones 3 and 4, respectively). These findings indicate that a group of dorsal tier cells in the spinal cord continued to express *Gbx2* from E8.5 to E12.5 while lower tier neurons down-regulated *Gbx2* between these stages along the R-C axis (Figure S3A–G). To assess how *Gbx2* progenitors marked at E9.5 regulated *Gbx2* expression as cells transitioned from the progenitor stage to early stages of differentiation, we again compared the *Gbx2* lineage (ß-gal) to *Gbx2*(GFP) expression at E12.5. Cells that had expressed *Gbx2*(GFP) at E9.5 co-expressed GFP at E12.5 in dorsal cord at all levels (Figures 2O, S4D) indicating that these cells continuously expressed *Gbx2* from E9.5–E12.5. In contrast, only a small amount of cells that had expressed *Gbx2* at E9.5 continued to express *Gbx2*(GFP) ventrally at the thoracic and lower limb levels at E12.5 (Figure 2O; arrow). Finally, we assessed *Gbx2* regulation in the cohort marked at E10.5. Cells in the ventral cord at all A-P levels that expressed *Gbx2*(GFP) at E12.5 were rarely derived from progenitors expressing *Gbx2*(GFP) two days earlier (Figure 2P, arrows and Figure S5D, S5E). However, *Gbx2*-derived cells marked at E10.5 were confined to the dorsal cord at all axial levels (Figure S5B,C) and persisted in their expression of *Gbx2*(GFP) over the ensuing two days (Figures 2P, S5D, S5E). Interestingly at E10.5, dorsal *Gbx2*-expressing cells (GFP+) were positioned in dl3–dl4, which is located in the middle third of the dorsal cord, and were not observed in dl1–dl2 (Figure 1K–1S). However, by E12.5 *Gbx2*(GFP)-expressing cells expanded and occupied the dorsal cord up to the roof plate at E12.5 (Figure 2K–L). This change in expression can be interpreted in two ways: Cells located dorsal to the *Gbx2*(GFP) expression domain at E10.5 began to newly express *Gbx2* after E10.5 or cells expressing *Gbx2*(GFP) at E10.5 migrated dorsally and settled near the roof plate. *In situ* hybridization with a *Gbx2* probe showed that *Gbx2*(GFP) expression at E10.5 was not in dl1–dl2. Coupled with GIFM at E10.5, these findings suggest that *Gbx2*-derived dl3–dl4 cells marked at E10.5 migrated dorsally and continued to express *Gbx2*(GFP) in their final location.

### Early neuronal fate decisions of the *Gbx2* lineage

**Figure 3:**
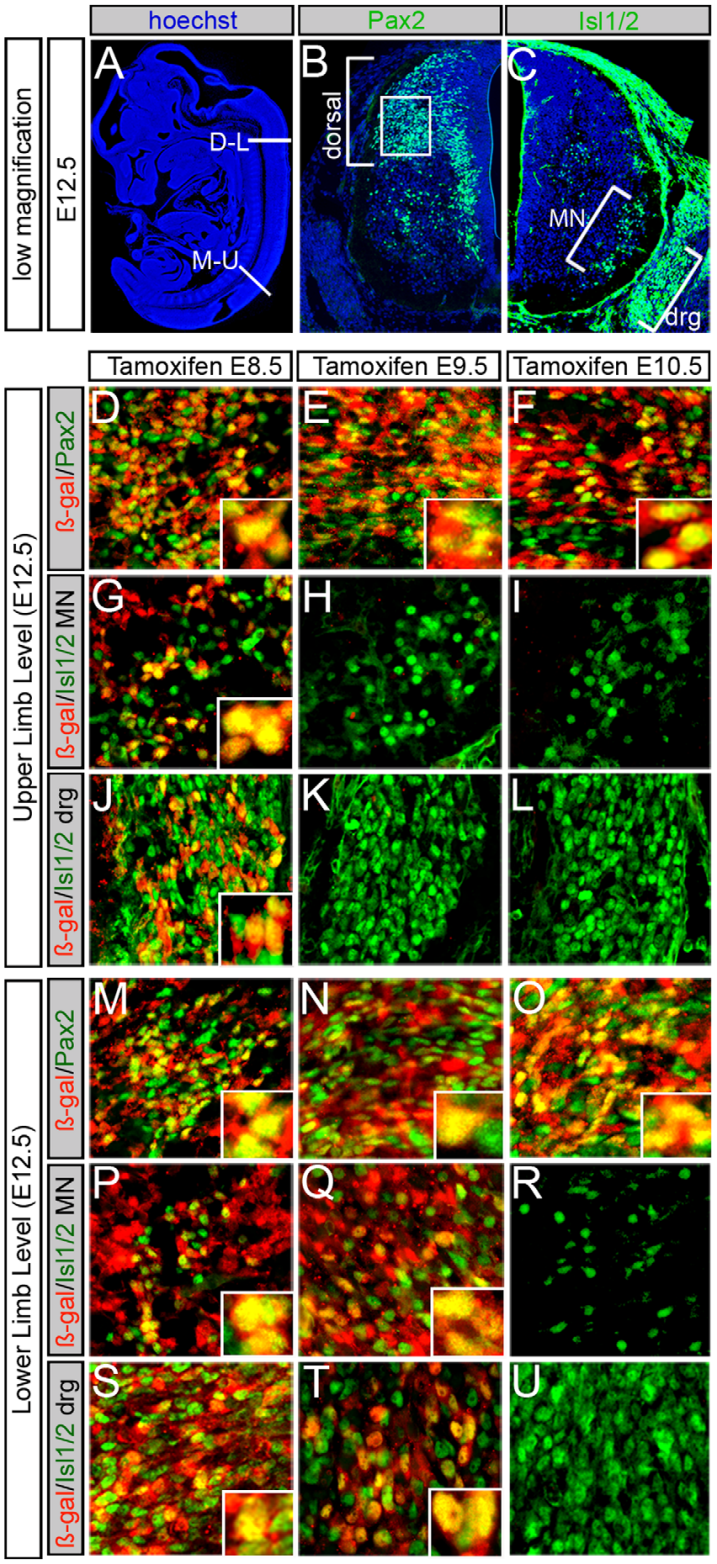
Molecular identity of the *Gbx2* lineage in E12.5 spinal cord. (A) Sagittal section of E12.5 embryo with nuclear staining (blue) showing regions analyzed. (B) Pax2 expression in dorsal spinal cord (indicated by bracket) in a hemi-transverse section. The box indicates the dorsolateral area of high magnification sampled for panels D–F, M–O (C) Isl1/2 expression in hemi-transverse sections of ventral spinal cord at the upper limb level. Isl1/2 is expressed in all developing motor neurons (MN) and dorsal root ganglia (DRG). Marker analysis of upper (D–L) and lower (M–U) limb levels at E12.5. The *Gbx2* lineage (ß-gal+, red) marked at E8.5, E9.5 or E10.5 gave rise to Pax2+ neurons (green) at both upper (D–F) and lower (M–O) limb levels; insets highlight colocalization. (G–I) The *Gbx2* lineage (ß-gal+, red) marked at E8.5, but not E9.5 or E10.5, contributed to ventral MNs (Isl1/2+, green) at upper limb level. (P–R) MNs (Is1/2+, green) at lower limb level were derived from the *Gbx2* lineage (ß-gal+, red) marked at E8.5 and E9.5, but not E10.5. (J–L) Neurons in upper limb DRG (Isl1/2+, green) were derived from the *Gbx2* lineage at E8.5 but not at later stages. (S–U) Caudal DRG (Isl1/2+, green) were derived from the *Gbx2* lineage at E8.5 and E9.5.

We next ascertained the intermediate fate of the *Gbx2* lineage marked at different stages by analyzing ß-gal+ cells and well-characterized markers at E12.5 (Figure 3A–U). Pax2, a transcription factor that distinguishes a subset of differentiating inhibitory dorsal horn interneurons (Figure 3B) [34] was expressed in *Gbx2*-derived cells marked at E8.5, E9.5, or E10.5 at upper (Figure 3D–F) and lower (Figure 3M–O) limb levels. Therefore, *Gbx2*(GFP)-expressing progenitors continually gave rise to dorsal Pax2+ inhibitory neurons. Because *Gbx2*(GFP) was expressed throughout the neural tube at E8.5 and contributed to the entire cord, we hypothesized that the *Gbx2* lineage would contribute to motor neurons at E12.5. Comparing ß-gal expression with Isl1/2 expression, which defines all differentiating motor neurons in ventral spinal cord [33], demonstrated that progenitors expressing *Gbx2*(GFP) at E8.5 gave rise to Isl1/2+ motor neurons at all A-P levels of the maturing spinal cord (Figure 3G,P). In contrast, *Gbx2*-derived progenitors marked at E9.5 no longer contributed to Isl1/2+ motor neurons at the upper limb level (Figure 3H), although the *Gbx2* lineage marked at E9.5 gave rise to motor neurons at the posterior lower limb level (Figure 3Q). We observed a complete exclusion of the *Gbx2* lineage marked at E10.5 from contributing to motor neurons at all A-P levels (Figure 3I,R). The dorsal root ganglia (DRG), which contain Isl1/2+ post-mitotic neurons [35], was derived from the *Gbx2* lineage (ß-gal+) marked at E8.5 along the full length of the maturing spinal cord (Figure 3J,S). Similar to motor neurons, ß-gal+ cells from E9.5 marking were not detected in upper limb DRG (Figure 3K), but were observed at lower limb level of spinal cord (Figure 3T). Finally, the *Gbx2* lineage marked at E10.5 did not contribute to Isl1/2+ DRG neurons at any A-P level (Figure 3L,U). Therefore, both dorsal and ventral populations in spinal cord are derived from a progenitors expressing *Gbx2* for twenty-four hours beginning at E8.5. Rapidly though, at the upper limb level, ventral motor neurons and DRG neurons were no longer derived from the *Gbx2* lineage. In contrast, motor neurons and DRG neurons at the lower limb level were derived from the *Gbx2* lineage twenty-four hours longer than those located rostrally.

### Terminal neuronal identity of the *Gbx2* lineage

**Figure 4:**
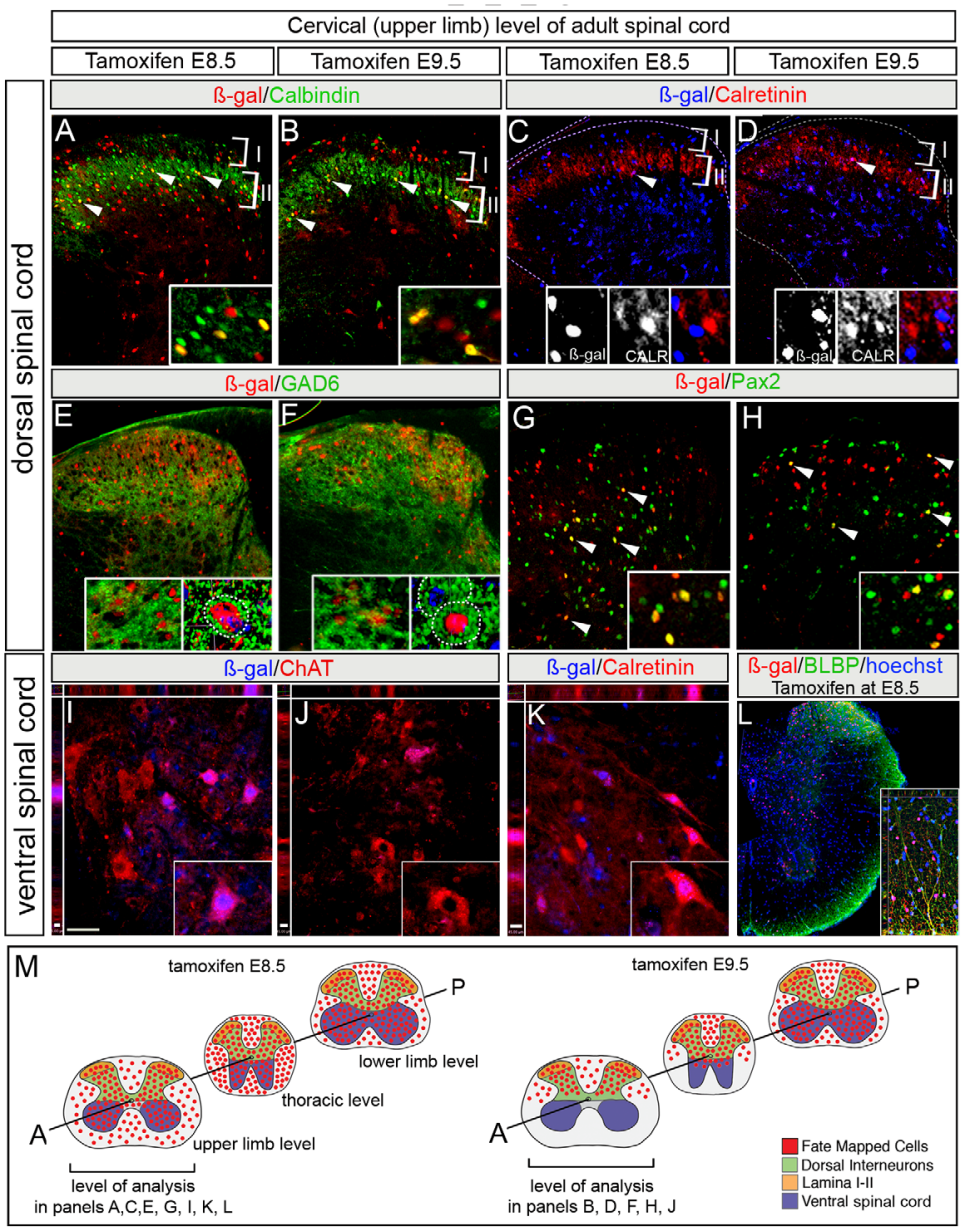
Terminal neuronal fate of the *Gbx2* lineage. The *Gbx2* lineage (ß-gal+, red) marked at E8.5 (A) or E9.5 (B) contributed to dorsal spinal cord. Calbindin+ interneurons (green) were derived from the *Gbx2*-lineage marked at both stages; Insets reveal colocalization. (C–D) *Gbx2*-derived cells (ß-gal+, blue) marked at E8.5 (C) or E9.5 (D) were interspersed and only rarely co-localize with calretinin+ (CALR) interneurons; Insets show lack of overlap in lamina II. (E–F) GABAergic inhibitory neurons (GAD6+, green) were derived from *Gbx2*-expressing progenitors marked at E8.5 (E) or E9.5 (F). Diffuse GAD6 labeling in axonal and dendritic projections engulfs ß-gal labeling in neuronal cell bodies (insets E–F). (G–H) *Gbx2*-derived cells (ß-gal+, red) marked at E8.5 (G) or E9.5 (H) contributed to Pax2+ (green) interneurons; arrowheads show co-localization. (I–K) Choline-Acetyl-Transferase (ChAT, red) or CALR (red) expression compared to ß-gal immunolabeling (blue) shows that the *Gbx2* lineage marked at E8.5 (I, K) but not E9.5 (J) contributed to both cholinergic motor neurons and interneurons in ventral horn at the upper limb level (Insets in I, K show colocalization; inset in J shows lack of contribution). (L) The *Gbx2* lineage (ß-gal+, red) marked at E8.5 contributed to brain lipid binding protein (BLBP)+ glial cells in white matter. (M) Summary schematic of *Gbx2* lineage (red circles) contribution to distinct laminae in the adult spinal cord. The summary is based on data presented in this figure and in Figure 6. The *Gbx2* lineage marked at E8.5 gave rise to motor neurons and interneurons in ventral spinal cord (blue) as well as dorsal lamina interneurons (orange). The *Gbx2* lineage marked at E9.5 occupied distinct D-V spinal cord domains depending on the A-P location in adult spinal cord. At the upper limb level (anterior, A), the *Gbx2* lineage marked at E9.5 gave rise to dorsal interneurons (green) including superficial lamina (orange), but not ventral motor neurons (blue). At the lower limb level (posterior, P), the *Gbx2* lineage marked at E9.5 spanned the D-V axis and gave rise to ventral motor neurons (blue) and dorsal interneurons (green, orange).

An important question in spinal cord development is how molecularly distinct progenitors contribute to adult spinal cord cytoarchitecture. To begin to address this, we marked the *Gbx2* lineage *in vivo* and ascertained the terminal fate of *Gbx2*-derived neurons marked at E8.5 and E9.5 using biochemical markers for functionally distinct neurons (Figure 4). Calbindin-D28K (CALB) and calretinin (CALR) are calcium-binding proteins that are expressed in a subset of interneurons located in superficial dorsal laminae I/II [36]. Lamina I was sparsely populated with CALB+ and CALR+ interneurons. In contrast, lamina II was densely packed with CALB+ interneurons and moderately populated with loosely arranged CALR+ neurons (Figure 4A–D) [37]. The *Gbx2*-lineage marked at E8.5 or E9.5 was evenly distributed across laminae I–II and co-localized with CALB+ in lamina II, but was only interspersed with CALB+ cells in laminae I (Figure 4A,B). In contrast, the *Gbx2*-lineage marked at E8.5 or E9.5 only rarely contributed to CALR+ neurons in layer II (Figure 4C,D). The *Gbx2*-lineage (ß-gal+) marked at E8.5 or E9.5 contributed to inhibitory neurons in dorsal laminae I–IV expressing glutamic acid decarboxylase (GAD) (Figure 4E,F, insets of 3-D rendered neurons provides clarity of labeling, which was confirmed with single 1 µm thick optical sections in the XZ and YZ plane, not shown). Finally, the *Gbx2* lineage marked at E8.5 and E9.5 gave rise to Pax2+ inhibitory interneurons dispersed throughout the dorsal horn (Figure 4G,H).

To determine whether *Gbx2*-derived Isl1/2+ motor neuron precursors marked at E8.5 terminally differentiated into motor neurons we compared ß-gal and Choline Acetyl Transferase (ChAT), which defines cholinergic motor neurons [38]–[40]. *Gbx2*-expressing progenitors marked at E8.5 gave rise to ChAT+ motor neurons at both cervical and lumbar levels of adult ventral spinal cord (Figure 4I,M). In contrast, *Gbx2*-descendants marked at E9.5 did not contribute to ChAT+ motor neurons at the cervical level (Figure 4J,M). This pattern of contribution with marking at E8.5, but not E9.5, was similar for ventral CALRET+ interneurons (Figure 4K,M). The *Gbx2* lineage was not confined to neurons as *Gbx2* fate mapping at E8.5, but not E9.5, resulted in ß-gal+/BLBP+ glial cells in the periphery of the ventral cord (Figure 4L). In summary, the dorsal *Gbx2* lineage marked at E8.5 and E9.5 comprise a heterogeneous population of CALB+, Pax2+, and GAD+ interneurons in the dorsal horn. The ventral *Gbx2* lineage contributed to both interneurons and motor neurons when marked at E8.5.

### *Gbx2* is required to pattern the neural tube and establish early spinal cord cytoarchitecture

**Figure 5:**
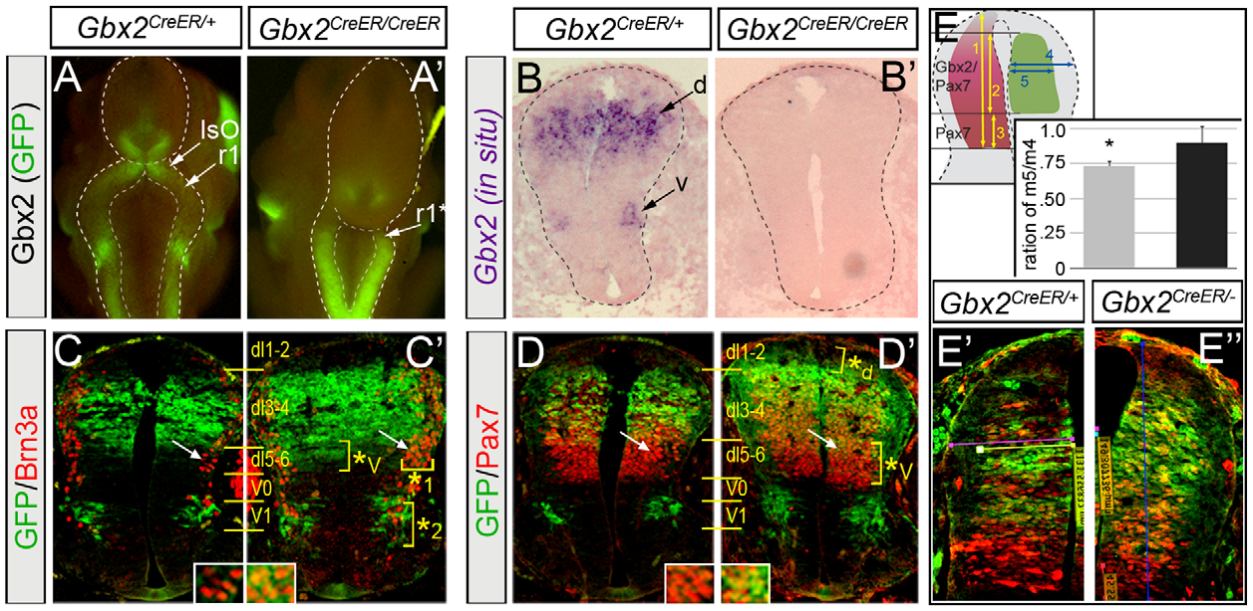
Distribution and identity of *Gbx2* mutant cells at E10.5. *Gbx2^CreER-ires-eGFP/+^* control heterozygote (A) and mutant *Gbx2^CreER-ires-eGFP/CreER-ires-eGFP^* embryos (A′) showing intact (r1) and reduced r1 (r1*), respectively at E10.5. *Gbx2 in situ* with an RNA probe on E10.5 transverse spinal cord sections from control (B) versus *Gbx2* mutant embryos (B′). Note that *Gbx2* was expressed in a broad dorsal (d) and in a restricted ventral (v) domain in wildtype embryos and absent in mutants. E10.5 control (C,D) and mutant (C′,D′) transverse sections immunolabeled with GFP (green) and indicated markers (red). *Gbx2*-deficient cells (GFP+, green) were co-localized with Brn3a in a wider swath of cells dorsally (*1). *Gbx2*(GFP) was broader and co-localized with Pax7 ventrally (*v) and in some cases dorsally (*d). Brn3a+ cells were in an ectopic ventral domain (*2); arrows indicate regions shown in insets. (E–E″) Compared to control *Gbx2* ^CreER-ires-eGFP/+^ sections (E′), those obtained from *Gbx2^CreER-ires-eGFP/−^* mutants (E″) showed a significantly broader *Gbx2*(GFP) medial-lateral domain∶spinal cord width (E, inset). Notably in E,E′ both embryos have only one copy of GFP.

To test whether *Gbx2* was required for spinal cord patterning, we bred *Gbx2^CreER-ires-eGFP/+^* heterozygotes [24] to yield *Gbx2^CreER-ires-eGFP/CreER-ires-eGFP^* homozygote embryos that were effectively *Gbx2*-null because the *CreER-ires-eGFP* cassette disrupts *Gbx2* in Exon 1 to generate a loss of function allele [24]. *Gbx2*-null mutant embryos had an obvious deletion of r1 consistent with previous reports of *Gbx2* mutants [21] (Figure 5A,A′). We assessed the expression of *Gbx2* by RNA *in situ* hybridization, which showed a *Gbx2* expression domain that did not expand to the lateral limit of the neural tube in controls and a complete absence of *Gbx2* transcripts in mutants (Figure 5B,B′). The knock-in configuration allowed us to detect cells that would have expressed *Gbx2* (*Gbx2*-null mutant cells) by EGFP whole mount fluorescence or GFP antibody labeling. *Gbx2^CreER-ires-eGFP/CreER-ires-eGFP^* mutants (n = 4) versus *Gbx2^CreER-ires-eGFP/+^* heterozygote controls (n = 3) revealed an apparent ventral expansion of *Gbx2*-mutant (GFP+) cells (Figures 5C,C′,*_V_). *Gbx2*-mutant (GFP+)/Pax7− cells were detected dorsal to the wildtype dl3–dI4 domain and often encroached upon dl1–2 (Figure 5D,D′,*_d_). *Gbx2*-mutant cells were also aberrantly positioned laterally and co-expressed Brn3a (Figure 5C′,*_1_), which was not seen in heterozygous controls (Figures 5C, arrow). The phenotype was also seen in the ventral spinal cord where ectopic Brn3a+ cells expanded ventrally and mixed with V0/V1 (Figure 5C,C′,*_2_). We were concerned that having two copies of *CreER* in *Gbx2^CreER/CreER^* mutants versus one copy in *Gbx2^CreER/+^* control littermates would confound the direct comparison of GFP expression in progenitors. Therefore, we repeated the experiment comparing *Gbx2^CreER/+^* (n = 3) to *Gbx2^CreER/−^* (n = 3) littermates, which allowed us to assess control versus mutant spinal cords, both with one copy of *GFP* (Figure 5E–E″). We measured the dorsal-ventral extent of GFP and although we observed a broader GFP domain in some *Gbx2^CreER/−^* mutants, it was not statistically significant. We also measured the medial to lateral extent of the GFP-expressing domain (figure 5E′,E″) and compared it to the medial to lateral extent of the spinal cord, which revealed a statistically significant medial expansion (P<0.05) (Figure 5E, inset graph).

**Figure 6:**
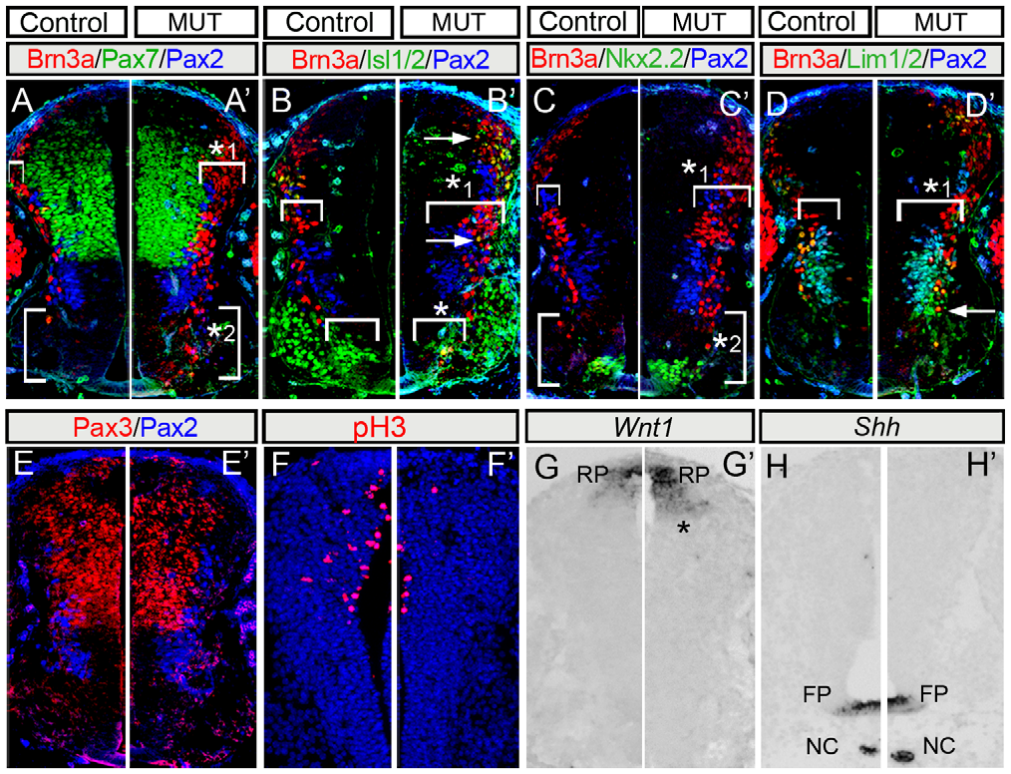
*Gbx2* loss affects spinal cord progenitor patterning. Hemi-transverse sections from E10.5 wildtype (A–H) and *Gbx2^CreER-ires-eGFP/CreER-ires-eGFP^* mutant embryos (A′–H′) triple immunolabeled with indicated markers. (A, A′) Broader Brn3a domain dorsally (*1) and Brn3a expressing cells in the ventral domain (*2) in *Gbx2* mutants. (B, B′) Brn3a+/Isl1/2+ neurons (arrows) and depletion of the medial-ventral domain of Isl1/2+ neurons (medial motor column,*) in *Gbx2* mutants. Qualitatively, some ventral Isl1/2+ neurons inappropriately expressed Brn3a (yellow overlap). (C,C′) Ectopic Brn3a expressing cells in close proximity to the ventral Nkx2.2 population (*2). (D,D′) Medial-lateral expansion of early differentiating neurons in *Gbx2* mutant embryos (*1, brackets). In addition, ectopic Brn3a+/Lim1/2+ neurons were seen ventral to their normal position (arrow). (E,E′) Pax3/Pax2 showing that the Pax3 domain is unchanged. (F,F′) Immunolabeling for phosphorylated-Histone H3 (pH3) showing fewer mitotic dorsally in mutant littermates. *Wnt1* expression in the roofplate (RP) was subtly expanded (G,G′,*) while *Shh* expression in the floor plate (FP) and notochord (NC) was unaffected in mutants (H,H′).

We then performed comprehensive triple marker analysis of cell types distributed along the D-V axis in *Gbx2^CreER/CreER^* mutants (n = 4) versus heterozygote controls (n = 3) at E10.5 (Figure 6). Dorsomedial progenitors expressing Pax7 and Pax3 were unperturbed in *Gbx2* mutants (Figure 6A,A′,E,E′). However, the domains defined by expression of Brn3a, Isl1/2, Pax2, and Lim1, and the combinatorial co-localization patterns between these markers appeared wider than normal in *Gbx2* mutants (Figure 6A–D′,*_1_) consistent with expanded *Gbx2*(GFP) expression. In control embryos, ventral Brn3a expression lined up with the ventral border of Pax2 and Lim1 expression and was juxtaposed to Isl1/2+ ventral motor neurons (Figure 6A,B,C,D). In contrast, *Gbx2* mutants had Brn3a+ cells that extended ventrally, almost reaching the Nkx2.2+ (V3) domain (Figure 6A′,C′,*_2_). In addition, *Gbx2* mutants had a reduction of ventromedial Isl1/2 motor neurons that comprise the median motor column (MMC) (Figure 6B,B′,*). To investigate a potential mechanism for the expansion of markers for differentiating neurons in the dorsal marginal zone, we stained for mitotic marker pH3 and observed a fewer mitotic cells in *Gbx2* mutants compared to heterozygous controls (Figures 6F,F′). Secreted WNT family molecules from the roof plate mediate patterning and control neuronal identity in dorsal spinal cord [41], while SHH signaling controls ventral cell fates [10]. Therefore, we evaluated *Wnt1* and *Shh* expression by *in situ* hybridization. The *Wnt1*-expression domain was subtly expanded in *Gbx2*-null mutants (Figure 6G,G′) while *Shh* remained unperturbed in (Figure 6H,H′). These findings suggest that the *Gbx2* mutant phenotype was largely independent of changes in opposing D-V morphogen gradients.

**Figure 7:**
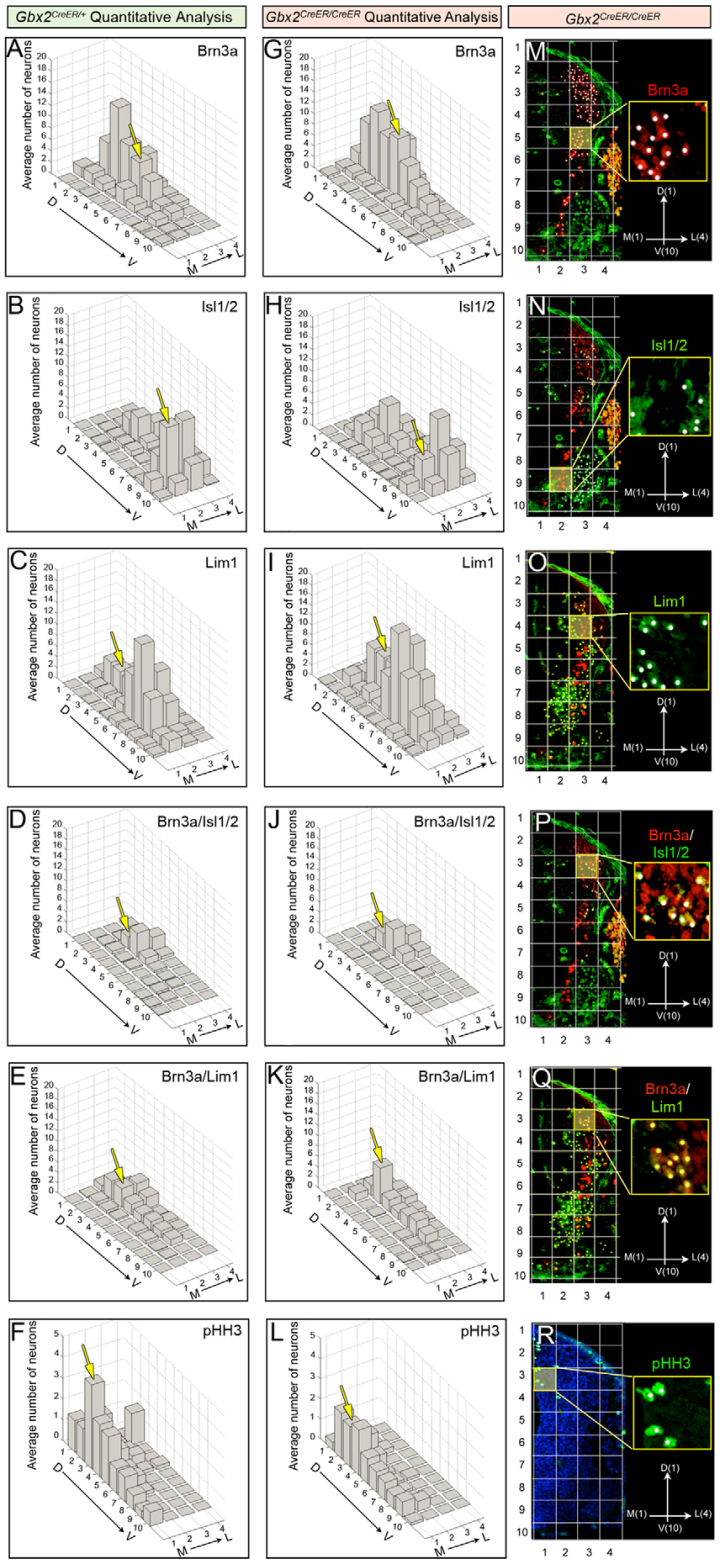
Quantitative assessment of aberrantly distributed spinal cord progenitors in *Gbx2* mutant embryos. Quantitative spatial analysis of control *Gbx2^CreER-ires-eGFP/+^* (A–F) and mutant *Gbx2^CreER-ires-eGFP/CreER-ires-eGFP^* (G–L) spinal cords at E10.5. The average number of progenitors was assessed by counting cells with expressing the indicated markers in two sections at the upper limb level from control embryos (n = 3) and mutant embryos (n = 4). To facilitate a clear comparison of the spatial distribution across samples, we a Cartesian coordinate system where ML_1_-DV_1_ represented the most medial-dorsal quadrant, ML_1_-DV_10_ the most medial-ventral quadrant, ML_4_-DV_1_ the most lateral-dorsal quadrant, and ML_4_-DV_10_ the most lateral-ventral quadrant (M–R). The yellow boxes in panels M–R are shown at higher magnification with white dots used to track counted cells. The yellow boxes also correlate with the domains that were highlighted in the graphs with a yellow arrow. Quantitative spatial mapping revealed the distribution of Brn3a+ cells (A,G,M), Isl1/2+ (B,H,N), Lim1+ (C,I,O), Brn3a+/Isl1/2+ (D,J,P), Brn3a+/Lim1+ (E,K,Q), and pHH3 (F,L,R).

To more fully assess the extent of the patterning defect in spinal cord progenitors, we first determined the total number of molecularly defined progenitors in hemi-transverse spinal cord (n = 3 controls, n = 4 mutants). There were no significant differences of the total number of indicated progenitors between controls (Figure 7A–F) and mutants (Figure 7G–L); see Quantitative approaches in Material and Methods for counts). We then generated quantitative spatial maps of the progenitors in control versus *Gbx2* mutants to assess the distribution of mutant progenitors (See Materials and Methods and Figure 7A–L). Our Cartesian coordinate system was comprised of 4 M-L columns (ML_1_–ML_4_, most medial to most lateral, respectively) and D-V rows (DV_1_–DV_10_, most dorsal to most ventral, respectively) (Figure 7M–R). In *Gbx2* mutant embryos at E10.5, there was a reduction of Brn3a+ progenitors along the D-V extent of the off-midline column (ML_2_), with the largest loss in dorsal ML_2_ (p<0.05), and an increase in Brn3a+ progenitors in ML_3_ (Figure 7A,G). We also observed an increase in Brn3a+ progenitors in the ventral half of the mutant spinal cord with the most prominent increase in ML_3_-DV_5_ to ML_3_-DV_9_ (Figure 7A,G). In control embryos, Isl1/2+ progenitors were primarily located in the MMC (ventral ML_2_) and LMC (ventral ML_3_) (Figure 7B). In *Gbx2* mutants, there was a significant depletion of Isl1/2+ progenitors in the MMC (p<0.05) (Figure 7H) and a significant increase of Lim1+ progenitors in MMC and LMC (Figure 7C,I). Double immunolabeling revealed a subtle increase Brn3a+/Isl1/2+ progenitors in domains DV_3_–DV_5_ in column ML_3_ in mutants versus controls and a reduction in Brn3a+/Isl1/2+ progenitors in MMC (p<0.05) (Figure 7D,J). Brn3a+/Lim1+ and Pax2+ cells were not significantly different between controls and mutants. Finally, there was a significant depletion of pHH3+ cells in mutant versus control (p<0.05) spinal cords (figure 7F,L). The decrease was seen across the extent of the cord with the largest loss observed in the dorsal half (DV_1_–DV_4_) of the spinal cord with an emphasis on the dorsal-lateral cord (ML_4_-DV_2_) (Figure 7F,L). This finding and the observation that the spinal cord was not overtly depleted of neurons suggests that proliferating cells prematurely exited the cell cycle and contributed to the general patterning defect resulting from *Gbx2* loss.

**Figure 8:**
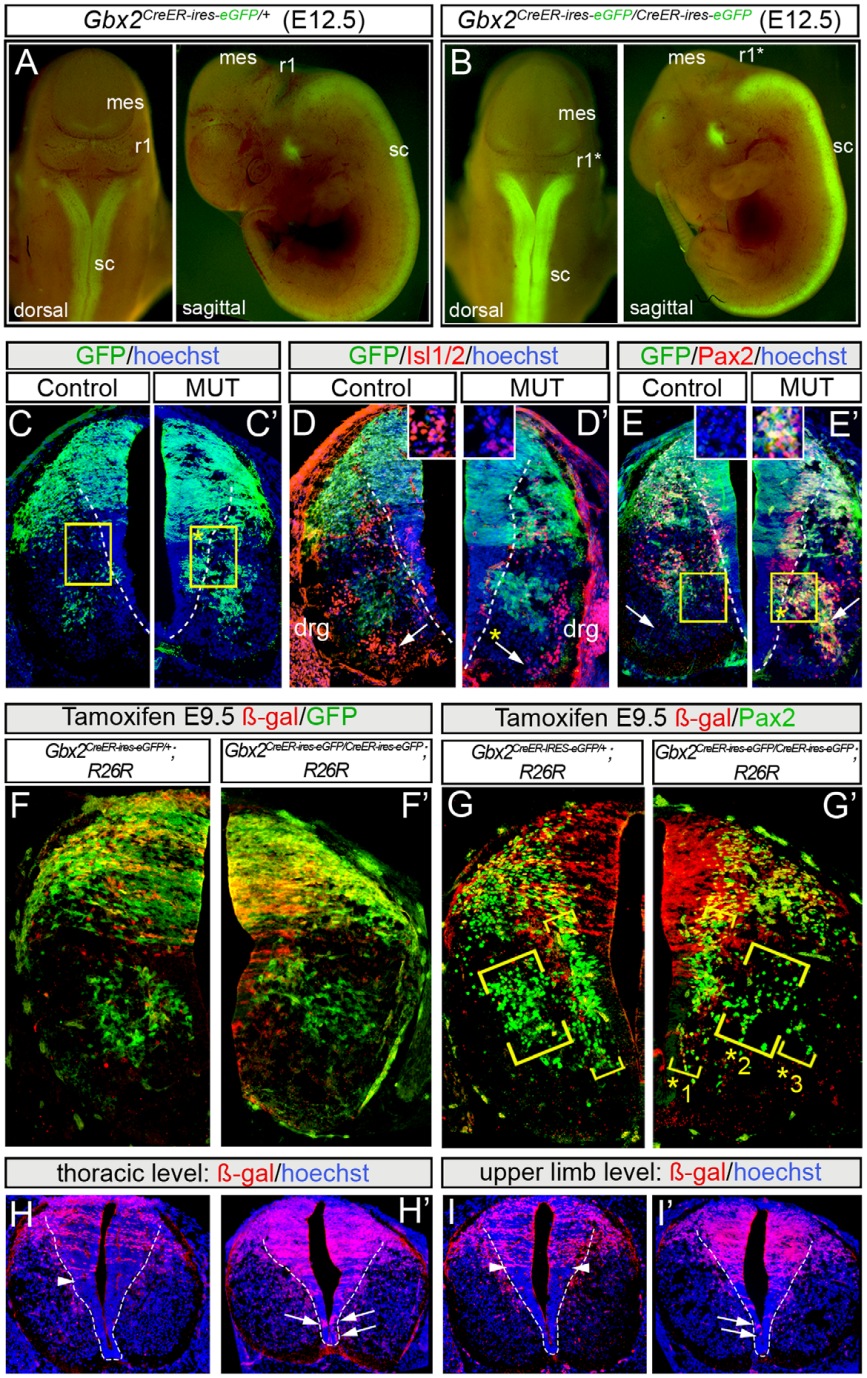
*Gbx2* mutant lineage at E12.5. Control *Gbx2^CreER-ires-eGFP/+^* (A) and mutant *Gbx2^CreER-ires-eGFP/CreER-ires-eGFP^* (B) embryos at E12.5; mutants have reduced r1 (r1*). (C,C′) GFP immunolabeling on level-matched hemi-transverse sections of E12.5 heterozygous control (C) versus *Gbx2* mutant embryos (C′). Ectopic clusters of *Gbx2* mutant (GFP+) cells (*) in the ventricular zone. (D,D′) Isl1/2 immunolabeling on transverse sections of E12.5 heterozygous (D) versus *Gbx2* mutant embryos (D′) showing loss of medial motor neurons in *Gbx2* mutant embryos (*). (E, E′) Immunolabeling for GFP and Pax2 showing ectopically located ventral *Gbx2*(GFP)-mutant/Pax2+ interneurons (*) in mutant embryos; arrows indicate regions shown in insets. (F–G′) GIFM of thoracic sections from wildtype control (F,G) versus mutant (F′,G′) spinal cord. (F) ß-gal and GFP immunolabeling showing the wildtype *Gbx2* lineage (ß-gal+, red) marked at E9.5 and *Gbx2*-expressing neurons at E12.5. (F′) The *Gbx2*-mutant lineage marked at E9.5 (ß-gal+, red) and *Gbx2*-mutants cells (GFP+, green) analyzed at E12.5. (G, G′) ß-gal expression resulting from lineage marking at E9.5 versus Pax2 expression in E12.5 control (G) versus *Gbx2* mutant embryos (G′). Note that some ventral Pax2+ cells are disorganized (*1, *2), and others are ectopically located (*3). (H–H′) Cells of the *Gbx2* mutant lineage marked at E9.5 reside in ectopic locations (arrows) that are ventral to the lineage boundary seen in controls (arrowheads).

*Gbx2^CreER-ires-eGFP/CreER-ires-eGFP^* mutants (n = 3) at E12.5 (Figure 8A,B) had *Gbx2*-mutant (GFP+) neurons that were distributed in a broader morphologically distinct dorsal ventricular zone replete with differentiating mutant neurons at E12.5 (Figure 8C,C′). This finding was consistent with our observations at E10.5. Dorsal Isl1/2+ interneurons and dorsal Pax2-expressing inhibitory interneurons were distributed in a similar pattern as controls (Figure 8D–E′). In contrast, we observed ectopic clusters of *Gbx2*(GFP) mutant cells in the ventricular and mantle zones of the ventral spinal cord (Figure 8C,C′,*). Ventromedial Isl1/2+ motor neurons of the MMC were depleted in *Gbx2* mutants (Figure 8D,D′,*) consistent with our findings at E10.5. The number of *Gbx2*(GFP) mutant cells that co-expressed Pax2+ in ventral spinal cord (33±12/hemisection) was doubled in comparison to wildtype *Gbx2*(GFP)+/Pax2+ cells (16±12/hemisection) (Figure 8E,E′,*). The lateral spinal cord of mutants had 2.5 times more *Gbx2*(GFP)+/Pax2+ cells while the medial spinal cord had a 1.8 fold increase. Finally, we addressed whether the state of mutant lineage derived cells changed over time by administering tamoxifen to *Gbx2^CreER-ires-eGFP/CreER-ires-eGFP^* embryos at E9.5. The *Gbx2*(GFP) mutant cells at E12.5 were not derived from the mutant progenitors (ß-gal+) marked at E9.5, which was similar to control littermates (Figure 8F,F′). Interestingly, the ventral Pax2+ neurons in *Gbx2^CreER-ires-eGFP/CreER-ires-eGFP^* embryos were scattered and loosely organized compared to controls (Figure 8G,G′,*_1_,*_2_,*_3_), but the Pax2+ cells were not derived from the *Gbx2* mutant lineage marked at E9.5. These findings suggested that a deficiency in *Gbx2* did not result in a cell-autonomous fate change in mutant progenitors marked at E9.5 (Figure 8G,G′). Rather, our findings indicated that a cohort of *Gbx2*(GFP)+/Pax2+ cells in mutant spinal cord resulted from an attempt to re-initiate the expression of *Gbx2*(GFP) at E12.5 and not from a failure of turning off *Gbx2*(GFP). Finally, the *Gbx2* mutant lineage was aberrantly distributed in ventricular zone of the ventral region of the spinal cord at thoracic and upper limb levels (Figure 8H–H′, arrows). This finding suggested that the lineage boundary was compromised in the absence of *Gbx2*.

## Discussion

The relationship between molecularly distinct progenitors that transiently or dynamically express specific genes during embryogenesis and the ultimate terminal differentiated cell types they give rise to is fundamentally important for the establishment of complex tissues. In addition, understanding developmentally regulated gene expression and cell lineage may be instructive for cell-based therapeutic approaches designed to replace specific populations that may be compromised in disease or injury. In this study, we used a combination of GIFM, *Gbx2* expression and marker analysis, and genetic mutant mice to examine the lineage and function of the transcription factor *Gbx2* at specific time points in spinal cord development. We chose *Gbx2* because the expression, contribution, and role of this transcription factor in spinal cord development has been unexplored.

### The *Gbx2* lineage in the ventral spinal cord is dynamically regulated and under tight temporal control

The dynamic change in *Gbx2* in ventral spinal cord over a short time window precludes understanding how progenitors expressing *Gbx2* at any given time are related to their spatial location and cell fate at later stages. To overcome this issue, we used GIFM to mark and track the *Gbx2* lineage. In addition, by comparing the *Gbx2* lineage (ß-gal+ cells) with respect to *Gbx2*(GFP) expression at E12.5 we determined how *Gbx2*(GFP) expression was regulated in the *Gbx2* lineage. The *Gbx2*-lineage marked at E8.5 is distributed throughout the entire ventral spinal cord consistent with its early broad expression. It is interesting that only a subset of the *Gbx2*-expressing cells in the ventral spinal cord (Gbx2^ventral^) continue to express *Gbx2*(GFP) at E12.5. This finding indicates that some *Gbx2* ^ventral^ cells at E12.5 initiated and maintained *Gbx2* expression beginning four days earlier while others did not maintain *Gbx2* expression (Figure S3). More importantly, this finding indicates that timing of gene expression within a genetic lineage defines distinct cohorts of neural progenitors. *Gbx2* ^ventral^ progenitors have a molecular identity of Gbx2+/Lim1/2+/Pax2+/Evx1/2−/Nkx2.2−/pH3− indicating that ventral *Gbx2*(GFP) expression was restricted to pV1 at this stage. The Gbx2^ventral^ population expanded between E10.5 and E12.5. suggesting that *Gbx2* expression is initiated *de novo* in a small number of Gbx2^ventral^ cells at E12.5. Interestingly, the *Gbx2*-lineage derived from *Gbx2* ^ventral^ progenitors at all axial levels at E10.5 did not significantly contribute to the *Gbx2*(GFP)-expressing population in ventral spinal cord at E12.5. Consistent with this, we very rarely detected *Gbx2*-derived cells marked at E9.5 in adult rostral-ventral spinal cord. One possibility to account for these observations is that Gbx2^ventral^ cells at E9.5 and E10.5 are post-mitotic and don't substantially expand during development, which masks their contribution. Coupled with the mosaic nature of GIFM [29], marking post-mitotic neurons may result in significantly fewer fate mapped cells than when marking a proliferating progenitor pool. A second possibility is that Gbx2^ventral^ cells marked at E9.5 or E10.5 are depleted over time or migrate into a more dorsal location. This seems unlikely because we do not see the appearance of loosely scattered cells emanating away from a diminishing ventral cohort of marked cells. Therefore, our data suggests that *Gbx2* expression is rapidly down-regulated in ventral progenitors that express *Gbx2* early (between E8.5 and E10.5) and that a small number initiate *Gbx2* late (between E10.5 and E12.5). Collectively, these mechanisms limit the contribution of *Gbx2* progenitors of the ventral spinal cord to distinct temporal epochs.

The dynamic regulation of *Gbx2*(GFP) ventrally occurs in a rostral-caudal direction that translates into a lineage code where V1 interneurons, Isl1/2+ motor neurons, and Isl1/2+ DRG neurons are temporally derived from the *Gbx2* lineage. The source of the *Gbx2* lineage contributing to DRG neurons are likely delaminating neural crest progenitors that express *Gbx2*, consistent with the role of *Gbx2* in patterning *Xenopus* [42], while motor neurons and V1 interneurons are derived from the neural tube. This is an interesting finding because it provides definitive evidence that interneurons and motor neurons in the ventral spinal cord are derived from the same genetic lineage at the same time. Subsequently, motor neurons and non-V1 interneurons derived from the *Gbx2*-lineage at E8.5 rapidly shut off *Gbx2*, while V1 interneuron progenitors remain *Gbx2+*. Our findings coupled with studies showing that molecular repression and response elements determine ventral spinal cord lineage decisions [8], [9], [17], [43] suggests that the molecular control of cell fate occurs in a common pool of *Gbx2*-expressing progenitors. Furthermore, our data suggest that the anterior-to-posterior down-regulation of *Gbx2* in the ventral neural tube helps define a short temporal window that delineates anterior motor neuron fate [*Gbx2*(GFP)+ cells marked at E8.5 only] versus V1 interneuron fate [*Gbx2*(GFP)+ cells marked from E8.5–E10.5]. In contrast, caudal Isl1/2+ motor neurons are derived from the *Gbx2* lineage, but over a prolonged two day window (marked from E8.5–9.5) while caudal V1 is marked from E8.5–E12.5, similar to anterior motor neurons. Interestingly, the caudal progenitors don't simply express *Gbx2* for a twenty four hour window that is offset compared to the rostral cord. This suggests that caudal motor neurons and DRG are comprised of progenitors that have a longer pulse of *Gbx2* expression. Collectively, these findings augment previous descriptions of rostral to caudal specification of motor neurons [44] and indicate that developmental timing and lineage as well as a transcription code identifies rostral versus caudal motor neurons and DRG neurons.

### The *Gbx2* lineage in dorsal spinal cord exhibits complex behaviors and contributes to inhibitory interneurons

In contrast to the ventral spinal cord, *Gbx2*(GFP) expression was apparent in the dorsal spinal cord (Gbx2^dorsal^) from E8.5–E12.5. After E8.5, *Gbx2*(GFP)-expressing cells co-expressed Pax7 dorsally in dl3/dl4 and by E10.5 Gbx2^dorsal^ cells had the following molecular identity: *Gbx2*(GFP)+/Pax3+/Pax7+. This is an interesting finding in the context of how dorsal interneurons are classified at E10.5. The absence of the transcription factor *Lbx1* distinguishes class A (dl1–dl3) interneurons from class B (dl4–dl6) interneurons that do express *Lbx1* [45]. Our data suggest that both class A dl3 and class B dl4 interneuron progenitors are derived from a common pool of progenitors. More importantly, though, our findings further advance our knowledge of defining progenitors in terms of anatomical position and marker analysis and can be used to refine progenitors based on genetic lineage. Therefore, dorsal interneurons can be classified as originating from the *Gbx2* ^dorsal^ lineage that is then further partitioned molecularly. *Gbx1* is expressed in a subset of class B progenitors referred to as the class B dlLA interneurons [22]. It is interesting to speculate that a combinatorial code of *Gbx* transcription factors is imparted on dorsal spinal cord progenitors to further refine a molecularly heterogeneous population of differentiating class B interneurons.

Our GIFM and marker analysis revealed cell behaviors of Gbx2^dorsal^ cells. Gbx2^dorsal^ cells at E10.5 are not localized to the Pax3+/Pax7− domain, which corresponds to dl1–dl2. However, by E12.5, Gbx2^dorsal^ cells were located in the dorsal spinal cord (excluding roofplate) and co-express Pax2, but not Isl1/2 consistent with an inhibitory interneuron identity. This suggests that either *Gbx2*-expression was initiated dorsally or that dl3–dl4 derived cells migrate dorsally between E10.5–E12.5. GIFM reveals that *Gbx2*-expressing progenitors initially located in dl3/dl4 at E9.5 and E10.5 contribute to a dorsal domain that spans 42.3±3.1% of the spinal cord, excluding the roof plate, at E12.5. Collectively, our findings are consistent with cells in dl4 migrating and settling in the dorsal horn [30], [45]. Interestingly, once *Gbx2*(GFP) expression is restricted dorsally, the marked lineage does not migrate into, or settle in, the ventral cord during development. In addition, the lineage derived cells continue to be retained in the adult dorsal cord. These findings indicate that a genetic lineage boundary is permanently fixed and confines the *Gbx2*-lineage to a dorsal compartment where they can intermingle and populate a broad dorsal domain. This is the first demonstration of a lineage boundary and compartmentalization in mouse spinal cord. A lineage restriction boundary is in agreement with clonal analysis in chick where clones labeled in the early neural tube disperse widely along the D-V axis, but are rapidly restricted from mixing and become confined to localized domains [46], [47]. Based on our timing of tamoxifen administration, the spinal cord lineage boundary in mouse is established at E9.5 rostrally and follows a rostral-to-caudal wave that completely fixes the boundary along the entire A-P axis within twenty-four hours. Collectively, we show that Gbx2^dorsal^ cells are confined to a dorsal compartment and acquire an inhibitory interneuron fate.

### *Gbx2* is required for correct spinal cord patterning

We utilized mice that have the *Gbx2* locus replaced by *CreER-ires-eGFP* and ascertained how the loss of *Gbx2* affects spinal cord development. The expression of GFP in *Gbx2^CreER-ires-eGFP/CreER-ires-eGFP^* cells shows that the *Gbx2* locus is capable of responding to gene regulation and that mutant cells do not die in the absence of *Gbx2*. Marker analysis revealed that *Gbx2*-mutant spinal cords had an improperly patterned ventral domain. This finding coupled with the early contribution of the *Gbx2* lineage (marked at E8.5, but not later) to the ventral spinal cord indicates an early role of *Gbx2* in patterning the ventral spinal cord. We also observed that *Gbx2* loss results in a dorsal patterning defect which consists of a broader than normal M-L domain of Brn3a, Isl1/2, Pax2, and Lim1/2 and a prominent decrease in mitotic cells in the dorsal spinal cord suggesting that *Gbx2* maintains the proliferative status of dorsal progenitors. Specifically, *Gbx2* may prevent progenitors from prematurely exiting the cell cycle and migrating laterally from the ventricular zone. This would be consistent with *Gbx2* involvement in cell proliferation during cerebellar development, where *Gbx2* loss results in decreased cell proliferation in the dorsal isthmus and medial cerebellar anlage [23]. In the context of cerebellar development, *Gbx2* indirectly maintains proliferation by inhibiting anti-proliferative activity of FGF8 [23]. However, whether *Gbx2* plays a direct cell-autonomous or non-autonomous role in spinal cord proliferation is unresolved.

We addressed whether the role of *Gbx2* in patterning is concomitant with a requirement in cell fate specification of interneurons. *Gbx2*(GFP)-mutant/Pax2+ precursors ectopically located in the ventral spinal cord at E12.5 were not derived from mutant *Gbx2*(GFP)-mutant progenitors marked at E9.5. This is interesting because the wildtype *Gbx2*-lineage marked at E8.5, but not at E9.5 or E10.5, gives rise to ventral Pax2+ cells (primarily at the dorsal margin of Pax2 expression) and the ventral Pax2+ cells generally do not continue to express *Gbx2* at E12.5. If *Gbx2*-mutant progenitors failed to down regulate the *Gbx2* locus between E8.5 and E9.5 and instead persisted in driving the expression of the *Gbx2* locus (and therefore CreER) at E9.5 and became Pax2+ precursors, then GIFM with tamoxifen at E9.5 would have marked these mutant cells. However, this was not the case, which indicates that the ectopic *Gbx2*(GFP)-mutant/Pax2+ cells were most likely Pax2+ interneurons that re-initiated *de novo* expression from the *Gbx2* locus (accounting for the persistent GFP expression) at E12.5. The dysregulation of the *Gbx2* locus and the patterning defect in the mutant spinal cord suggests that spinal cord gene regulation is altered in the absence of *Gbx2*. This is consistent with the structure and function of *Gbx2*, which acts as a transcriptional repressor that uses an Engrailed-like homology 1 motif to interact with the WD40 domain of the *Groucho/Tle* family of co-repressors [48]. Interestingly, this is a typical feature of transcription factors that are used for cross repressive interactions in the spinal cord [43] and *Gbx2* uses this mechanism to repress *Otx2* expression during midbrain-hindbrain development [18]. It will be interesting in future studies to test if Gbx2 uses this mechanism in spinal cord patterning.

*Gbx2* establishes lineage boundaries that demarcate thalamic nuclei [24] and positions the midbrain/anterior hindbrain gene expression interface [20], which is also a lineage boundary [25]. GIFM shows that the cells derived from the mutant *Gbx2* lineage are dispersed in the ventral ventricular zone violating the boundary. This finding indicates that similar to lineage boundaries in thalamus, the spinal cord lineage boundary requires *Gbx2* for its establishment or maintenance. We also show that loss of *Gbx2* results in a small ventral expansion of *Wnt1* expression in the roof plate at E10.5. *Gbx2* does not abut the roofplate at this stage suggesting an indirect mechanism underpinning *Wnt1* expansion. Previous studies using electroporation to ectopically express *Wnt1* throughout the entire dorsal half of the cord in chick increases dl2–dl4 neurons [11]. Additionally, *Wnt1/Wnt3a* double knockouts have dorsal spinal cord patterning defects [41]. Collectively, these findings suggest that increased WNT signaling may contribute to the dorsal *Gbx2* mutant phenotype.

### Long term lineage analysis links timing of *Gbx2* expression in progenitors to terminal interneuron fates

The adult spinal cord is comprised of molecularly distinct interneurons that have a laminar cytoarchitectonic organization [1]. However, whether genetic history and developing timing impact this organization is not well understood. In the ventral spinal cord the *Gbx2* lineage gives rise to ventral interneuron progenitors and to V1, but only during a short time frame. These progenitors later become CALR+ interneurons in the ventral spinal cord. It has been shown previously that V1 interneurons with a history of expressing *En1* primarily become CALB+ but not group 6 or group 7 CALR+ interneurons [49]. Collectively, these findings suggest that *En1* and *Gbx2* contribute to unique classes of interneurons. In support of this idea, *En1* expression is limited to a subset of V1 interneurons during embryonic development [50] and we show that *Gbx2*(GFP) is also present in a subset of V1 interneurons.

In contrast, the Gbx2 lineage, which becomes confined to the dorsal spinal cord by a lineage restriction boundary, contributes to interneurons in dorsal superficial lamina II when marked at both E8.5 and E9.5. The *Gbx2* lineage-derived interneurons express CALB, but only rarely CALRET, and also gives rise to GAD+ GABAergic inhibitory neurons in the adult dorsal spinal cord. Finally, Pax2+ dILA class B late born interneurons [22], [34], which are derived from the *Gbx2*-lineage marked from E8.5–E10.5 continue to express *Gbx2* at E12.5 and contribute to Pax2+ neurons in the adult. These findings are consistent with Pax2 being expressed in, and required for, GABAergic neuron development [34]. These findings also show that *Gbx2*-derived dorsal spinal cord interneuron progenitors are not progressively restricted in competence. Thus, dorsal spinal cord interneurons are not generated in a similar manner as *Drosophila* neuroblasts [51], [52]. Interestingly, cortical interneurons derived from the *Nkx6*-2 lineage also do not follow a progressive restriction in competence model [53], but rather behave similarly to the manner in which spinal cord interneurons are established. In summary, our studies forge a relationship between molecularly distinct progenitors that dynamically express *Gbx2* during embryogenesis and the ultimate terminal differentiated neuronal types they give rise to in the adult. We also revealed complex cellular and genetic events underpinning spinal cord development and progenitor-to-neuron relationships.

## Materials and Methods

### Ethics Statement

Mice were housed and handled in accordance with Brown University Institutional Animal Care and Use Committee (IACUC) guidelines. The Brown University IACUC reviewed and approved this study (IACUC #0909081, approved October 28, 2009) in accordance with the OLAW PHS Policy on the Humane Care and Use of Laboratory Animals and Animal Welfare Assurance (#A3284-01). The ACF operation is overseen by the Brown University Animal Care and Use Committee and is accredited by AAALAC.

### Genetic Knock-In Mice, Reporters, and Mutant Mice

*Gbx2^CreER-ires-eGFP^* was generated by replacing one allele of *Gbx2* with *CreER^T2^-ires-eGFP* [24]. We bred *Gbx2^CreER-ires-eGFP^* ^/+^ heterozygote knock-in mice (gratefully obtained from James Li, U. Conn, Health Center) to mice containing the *R26R* reporter allele [54] to generate *Gbx2^CreER-ires-eGFP^*;*R26R* mice that were bred with the wild type swiss webster strain mice (Taconic Farms) to maintain *Gbx2^CreER-ires-eGFP/+^;R26R* progeny. We identified double positive progeny with primers recognizing *CreER* and *R26R* as previously described [26]. *Gbx2* heterozygotes were phenotypically indistinguishable from *Gbx2^+^* ^/+^ wildtype mice [21] and RNA *in situ* hybridization analysis confirmed that EGFP expression faithfully recapitulated endogenous *Gbx2* expression (See text for details). We bred *Gbx2^CreER-ires-eGFP^* ^/+^ heterozygote males and females to yield both *Gbx2^CreER-ires-eGFP^* ^/*+*^ heterozygote control embryos (n = 3) and *Gbx2^CreER-ires-eGFP^* ^/*CreER-ires-eGFP*^ homozygote embryos (n = 4) that lack *Gbx2* and instead express two copies of *CreER*-IRES-*eGFP*. Homozygote Gbx2-deficient embryos were easily discernible by phenotype analysis because they exhibit a severe reduction of r1 [20], [21], [23] and strikingly increased whole mount EGFP fluorescence compared to heterozygotes. *Gbx2^CreER-ires-eGFP^* ^/*/+*^ control heterozygous embryos (n = 3) *Gbx2^CreER-ires-eGFP^* ^/*/−*^ (n = 3) null mice were generously provided by J. Li. The loss of *Gbx2* in embryos was confirmed for their lack of *Gbx2* expression compared to heterozygous embryos by RNA *in situ* hybridization.

### Genetic Inducible Fate Mapping

Fate mapping procedures were done as previously described [26], [27], [55]. Vaginal plugs detected at 9–10AM in the morning after mating were considered E0.5 days post-conceptus. Tamoxifen was dissolved in corn oil at a final concentration of 20 mg/ml. To induce GIFM, 0.2 ml of tamoxifen-corn oil solution (4 mg tamoxifen) was administered by oral gavage at 9AM on day E8.5, E9.5, or E10.5 to pregnant swiss webster females that had mated with *Gbx2^CreER-ires-eGFP/+^; R26R* males. GIFM could be performed on the *Gbx2*-null background by administering tamoxifen to pregnant Gbx2*^CreER-ires-eGFP^* ^/+^; *R26R* females that had mated with *Gbx2^CreER-ires-eGFP/+^; R26R* males. Mouse embryos were analyzed at the stages indicated in the text (n≥4 across two different litters); adult mice were analyzed at 3 months of age (n = 4 across two different litters).A balanced one-way analysis of variance power calculation (power.anova.test) was done to determine appropriate sample sizes. We held constant the statistical p value that meets our requirement for significance (p = 0.05), as well as the within and between group variance (set at 1 and 1.5, respectively). We varied the sample (number of animals/group) size and calculated the power. Sampling four animals/group resulted in a confidence interval of 80%, which is consistent with our previously published analyses [55], and is acceptable to assess statistical significance.

### Immunocytochemistry, X-gal Histochemistry, RNA *In Situ* Hybridization

Experimental details of tissue processing and techniques have been described in detail [26] and can be downloaded from http://research.brown.edu/myresearch/Mark_Zervas. Embryos were photographed for wholemount EGFP fluorescence to visualize *Gbx2*-expressing domains, then fixed in 4% paraformaldehyde, cryoprotected with 15% and 30% sucrose, embedded in OCT compound, and cryosectioned at 12–16 micron thickness depending on the stage. Adult spinal cord tissue was fixed by inter-cardiac perfusion of 10 ml PBS followed by 15 ml of 4% formaldehyde. After dissecting the intact adult spinal cord and brain, tissue samples were allowed to sit in 4% PFA for at least one night, cryoprotected, OCT embedded, and cryosectioned at 20–25 microns. The following antibodies were used: rabbit anti-GFP 1∶600 (Invitrogen), rat anti-GFP 1∶500 (Invitrogen), goat anti-ß galactosidase 1∶500 (Biogenesis), chick anti-ß galactosidase 1∶500 (Abcam), mouse anti-Pax7 1∶20 (Developmental Studies Hybridoma Bank), mouse anti-Lim1/2 1∶50 (DSHB), mouse anti-Isl1/2 1∶50 (DSHB), mouse-anti Pax6 1∶500 (DSHB), rabbit-anti Pax2 1∶50 (Zymed), mouse anti-Pax3 1∶50 (DSHB), mouse anti-Evx1/2 1∶50 (DSHB), mouse anti-Nkx2.2 1∶50 (DSHB), rabbit anti-Brn3a 1∶1000 (gift of Eric Turner), guinea pig anti-Brn3a 1∶500 (gift of Eric Turner), mouse anti-GAD6 1∶50 (DSHB), goat anti-ChAT 1∶100 (Chemicon), rabbit anti-phosphorylated histone H3 1∶200 (Chemicon), goat anti-Calbindin 1∶1000 (Swant). The appropriate secondary antibodies were used at 1∶500 dilutions from Invitrogen-Molecular Probes or Jackson Immunoresearch. Images were captured on an epifluorescent compound microscope (Leica DMB600) and processed using Volocity imaging software (Improvision). ß-gal histochemistry was performed on freshly perfused adult spinal cord tissue samples by immersing samples in X-gal (5-Bromo-4-Chloro-3-Indolyl-beta-D-galactopyranoside) substrate solution over night. RNA *in situ* hybridization was performed with labeled anti-sense probes to *Gbx2*, *Shh*, and *Wnt1* according to published protocols [25], [26].

### Quantitative approaches

Embryos were sectioned in the transverse orientation and immunolabeled with the markers indicated above and in the text. The number of cells was counted on the right side of the transverse sections. Marker positive cells were counted and analyzed from 2 sections from each of 4 mutant and 3 control embryos (Figures 6 and 7). Control spinal cords at E10.5 had the following counts (average ± standard deviation/hemisection): Brn3a+ (80±40), Isl1/2+ (86±43), Lim1/2+ (98±52), Brn3a+/Isl1/2+ (18±9), Brn3a+/Lim1+ (27±16), Pax2+ (63±23). *Gbx2^CreER/CreER^* mutants had the following counts: Brn3a (87±29), Isl1/2 (82±35), Lim1 (135±68), Brn3a+/Isl1/2+ (17±7), Brn3a+/Lim1+ (27±8), Pax2+ (71±19). To facilitate a clear comparison of the spatial distribution across samples, we applied a grid [composed of 4 M-L columns (ML_1_–ML_4_) and 10 D-V rows (DV_1_–DV_10_) to give a 4×10 matrix] to one half of the spinal cord as shown in (Figure 7M–R). With this Cartesian coordinate system, position ML_1_-DV_1_ represented the most medial-dorsal quadrant, ML_1_-DV_10_ the most medial-ventral quadrant, ML_4_-DV_1_ the most lateral-dorsal quadrant, and ML_4_-DV_10_ the most lateral-ventral quadrant. The number of marked cells in each quadrant was averaged across all animals of the same genotype and standard deviations were calculated. Shapiro-Wilks tests for normality were applied to the data and ANOVAs were used to determine if there was a significant difference between the number of Brn3a, Isl, Lim, Brn3a/Isl, Brn3a/Lim or pHH3 positive cells in the mutant versus control animals p values are indicated in the text. We compared extent of the *Gbx2*(GFP) domain in *Gbx2^CreER/+^* (n = 3) to *Gbx2^CreER/−^* (n = 3) littermates, which allowed us to assess control versus mutant spinal cords, both with one copy of *GFP* (Figure 5E). We measured the dorsal-ventral extent of GFP (measurement 2) in relation to the dorsal marker Pax7 (measurement 1) and calculated the ratio of GFP to Pax7 (m2/m1). We also measured the medial to lateral extent of the GFP-expressing domain (measurement 5) and compared it to the medial to lateral extent of the spinal cord (measurement 4) and calculated this ratio (m5/m4) (Figure 5E, inset). Finally, we counted the number of *Gbx2*(GFP)/Pax2 expressing cells in control versus mutants (two transverse hemisections/embryo).

## Supporting Information

Figure S1 **GFP and RNA** ***in situ*** **hybridization analysis.** (A, B) Adjacent transverse sections from E12.5 embryos labeled with a GFP antibody (A) or with a *Gbx2* RNA probe showing expression patterns that were identical. Note that *Gbx2* and GFP were excluded from the roof plate (rp) and dorsal root ganglia (drg), but were distributed in a bilateral broad dorsal regions (top bracket) and in smaller bilateral ventral domains (lower bracket). Gbx2 transcripts appeared to be more intense in the marginal zone compared to the ventricular zone although GFP in immunolabeled sections was more uniformly seen in both zones; this is also true for the deep dorsal region of the spinal cord. This may reflect the sensitivity of the antibody/immunolabeling versus anti-sense probe/*in situ* hybridization. In addition, the levels of GFP transcripts/protein are unlikely to mimic the levels of *Gbx2* transcripts/protein due to differential processing. We were not suggesting that the levels of GFP correlate with *Gbx2*, but rather that GFP was a reliable indicator of cells expressing *Gbx2*.
(TIF)

Figure S2 **Schematic of GIFM strategy.** (A) The allelic combinations required for GIFM are shown. *Gbx2^CreER-ires-eGFP^* encodes for *EGFP* and allows for cells expressing *Gbx2* at the time of analysis (GFP+) to be identified, while *CreER* encodes a tamoxifen-regulated *Cre* for lineage analysis. The recombined ‘marking’ of cells is achieved with the *R26R* reporter allele. (B–E) GIFM strategy. All cells in *Gbx2^CreER-ires-eGFP^;R26R* embryos contain the reporter allele (shown in the nucleus), but which is quiescent because of a *loxP* (white triangles) flanked *Stop* cassette (red line). *Gbx2*-expressing cells in the hindbrain and spinal cord (GFP+, green ovals) express CreER protein (large green oval), which is sequestered away from the nucleus and confined to the cytoplasm because of the interaction with hsp90 (orange oval). (C) Upon tamoxifen (red T) administration, CreER is released from hsp90 freeing it to translocate to the nucleus. (D) Once in the nucleus, CreER mediates recombination between same-site orientation *loxP* sites causing the deletion of the intervening *Stop* cassette. This results in the LacZ reporter being turned on. Notably, this event occurs for approximately 24–30 hours *in vivo* because of tamoxifen pharmacokinetics [56]. (E) The recombined reporter allele is constitutively expressed, heritable, and highly reproducible and serves as a genetic lineage tracer. The fate mapped *Gbx2* lineage (blue ovals) can then be followed over time even after *Gbx2* expression is extinguished.
(TIF)

Figure S3 **Gbx2 lineage marked at E8.5 broadly contributes spinal cord at E12.5.** (A–G) ß-Gal and GFP antibody labeling on sagittal sections of an E12.5 embryo marked by GIFM at E8.5. Two cell populations defined by *Gbx2* lineage were distinguished at E12.5: (1) The cells that were *Gbx2*(GFP)^+^/ß-Gal^+^ at E12.5 had continuously expressed Gbx2 from E8.5–E12.5 in spinal cord. These cells were distributed in the broad dorsal domain and in a restricted ventral domain along the full rostral-caudal extent of cord. (2) The cells that expressed *Gbx2* at E8.5, but no longer expressed *Gbx2* (*Gbx2*(GFP)^−^/ß-Gal^+^) at E12.5 were observed ventrally and dorsally along the full length of cord. The *Gbx2* gene was turned off in these cells at some point between marking and E12.5 (H–J) ß-Gal antibody labeling of cells marked by GIFM at E8.5 versus antibody labeling to Pax2 on sagittal sections of an E12.5 embryo. Colocalization revealed that *Gbx2*(GFP) expressing cells marked at E8.5 gave rise to dorsal and ventral Pax2+ cells at all R-C levels; arrowheads show examples of co-localization. Sections shown here are from a medial plane, which precludes seeing dorsal Pax2+ cells because they are distributed in a “V-shaped” distribution (See Figure 1). (L–P) The *Gbx2* lineage (ß-Gal+) marked at E8.5 by GIFM versus antibody labeling of Isl1/2+ cells. Colocalization revealed that *Gbx2* expressing cells marked at E8.5 gave rise to dorsal and ventral Isl1/2+ cells at all R-C levels.
(TIF)

Figure S4 **Gbx2 lineage marked at E9.5 makes varied contributions to the E12.5 spinal cord at different rostral caudal positions.** (A) A lateral view of an E12.5 mouse embryo analyzed by wholemount *Gbx2*(GFP) fluorescence revealed *Gbx2* expression. (B–E) GIFM by tamoxifen administration at E9.5 and analysis at E12.5 (B,C) Cells expressing *Gbx2* at E9.5 and marked by GIFM that were detected by ß-Gal on sagittal sections gave rise to cells in dorsal cord at rostral levels (upper limb level) while at caudal levels (lower limb), the *Gbx2* lineage gave rise to cells distributed across the full dorsal-ventral and medial-lateral extent of cord. (D) ß-Gal and GFP antibody labeling on transverse sections of cord at the upper limb level. Cells in the dorsal domain that were GFP^+^/ß-Gal^+^ continuously expressed *Gbx2* from E9.5–E12.5. Cells in the ventral domain that were GFP^+^/ß-Gal^−^ did not express *Gbx2* at E9.5 but expressed *Gbx2* at E12.5. (E) ß-Gal and Pax2 immunolabeling on transverse sections at the upper limb level of an E12.5 embryo revealed that *Gbx2* expressing cells marked at E9.5 give rise to dorsal Pax2^+^ cells but only rarely to ventral Pax2^+^ cells. (F) ß-Gal and Isl1/2 immunolabeling on transverse sections showed marked cells were interspersed with dorsal Isl1/2^+^ cells but did not give rise to dorsal or ventral Isl1/2^+^ cells.
(TIF)

Figure S5 **Gbx2 lineage marked at E10.5 contributes to dorsal spinal cord at E12.5.** (A) A lateral view of an E12.5 mouse embryo analyzed by *Gbx2*(GFP) wholemount GFP fluorescence showed *Gbx2* expression. (B,C) Cells that expressed *Gbx2* at E10.5 were detected by ß-Gal immunolabeling on sagittal sections of rostral spinal cord. The *Gbx2* lineage marked at E10.5 gave rise to a tightly restricted dorsal domain at the upper (B) and lower (C) limb level. (D,E) ß-Gal and GFP antibody labeling on thoracic level transverse sections. Most cells in the dorsal domain were *Gbx2*(GFP)^+^/ß-Gal^+^ and therefore had continuously expressed *Gbx2* from E10.5–E12.5. There was no ß-Gal expression in ventral cord at the thoracic level. Cells in the ventral domain that were *Gbx2*(GFP)^+^/ß-Gal^−^ did not express *Gbx2* at E10.5, but did express *Gbx2* at E12.5. (F,G) ß-Gal and Pax2 immunolabeling on thoracic level transverse sections revealed that *Gbx2* expressing cells marked at E10.5 gave rise to dorsal Pax2^+^ cells but not ventral Pax2^+^ cells. (H,I) ß-Gal and Isl1/2 immunolabeling on thoracic level transverse sections were interspersed with dorsal Isl1/2^+^ cells but did not give rise to dorsal or ventral Isl1/2^+^ cells.
(TIF)

Figure S6 **Distribution of** ***Gbx2*** **lineage marked at E8.5 and E9.5 in adult spinal cord.** (A–B) Sagittal view of whole mount brain and spinal cord processed for X-gal histochemistry show the *Gbx2* lineage marked at E8.5. Panels A and B show medial and lateral CNS, respectively. (C–E) The *Gbx2* lineage marked at E8.5 was detected on transverse hemi-sections of adult spinal cord at the cervical enlargement (upper limb level, C), thoracic flank level (D), and lumbar enlargement (lower limb level, E). The *Gbx2* lineage (ß-Gal^+^) contributed to the full extent of the D-V spinal cord along the entire A-P axis including both grey and white matter (E). (F–H) The *Gbx2* lineage (ß-gal+) marked at E9.5 at the upper limb (F), thoracic flank (G), and lower limb (H) levels. The *Gbx2* lineage was restricted dorsally at the upper limb level but contributed to both dorsal and ventral domains at the lower limb levels although the contribution to white matter was less than when marked at E8.5. (I–J) Sagittal whole mount views of entire brain and spinal cord with X-gal histochemical staining showing cells marked at E9.5. Panels I and J show, respectively, medial and lateral CNS. Insets in panels B and J show DRG (arrows).
(TIF)
