# Supplementary figures and images for: The Lineage Contribution and Role of Gbx2 in Spinal Cord Development
Source: PLoS One. 2011 Jun 16;6(6):e20940. doi: 10.1371/journal.pone.0020940 (PMC3116860; doi:10.1371/journal.pone.0020940)

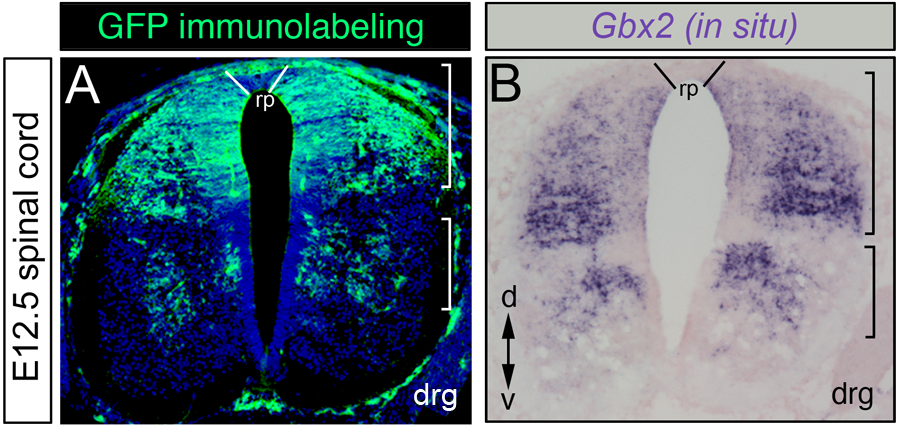

Supplement: Figure S1 — GFP and RNA in situ hybridization analysis. (A, B) Adjacent transverse sections from E12.5 embryos labeled with a GFP antibody (A) or with a Gbx2 RNA probe showing expression patterns that were identical. Note that Gbx2 and GFP were excluded from the roof plate (rp) and dorsal root ganglia (drg), but were distributed in a bilateral broad dorsal regions (top bracket) and in smaller bilateral ventral domains (lower bracket). Gbx2 transcripts appeared to be more intense in the marginal zone compared to the ventricular zone although GFP in immunolabeled sections was more uniformly seen in both zones; this is also true for the deep dorsal region of the spinal cord. This may reflect the sensitivity of the antibody/immunolabeling versus anti-sense probe/in situ hybridization. In addition, the levels of GFP transcripts/protein are unlikely to mimic the levels of Gbx2 transcripts/protein due to differential processing. We were not suggesting that the levels of GFP correlate with Gbx2, but rather that GFP was a reliable indicator of cells expressing Gbx2. (TIF) [file pone.0020940.s001.tif]

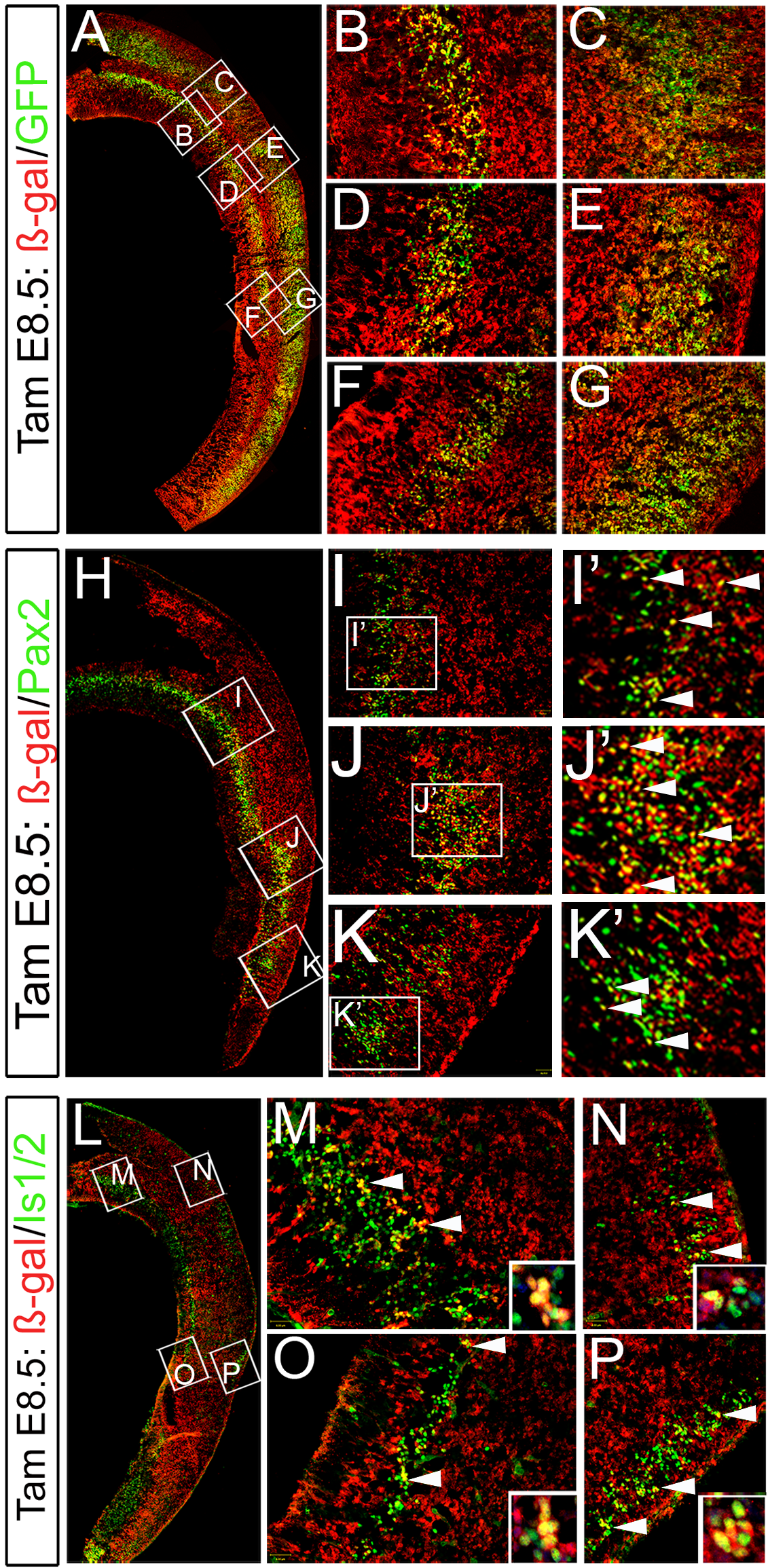

Supplement: Figure S3 — Gbx2 lineage marked at E8.5 broadly contributes spinal cord at E12.5. (A–G) ß-Gal and GFP antibody labeling on sagittal sections of an E12.5 embryo marked by GIFM at E8.5. Two cell populations defined by Gbx2 lineage were distinguished at E12.5: (1) The cells that were Gbx2(GFP)+/ß-Gal+ at E12.5 had continuously expressed Gbx2 from E8.5–E12.5 in spinal cord. These cells were distributed in the broad dorsal domain and in a restricted ventral domain along the full rostral-caudal extent of cord. (2) The cells that expressed Gbx2 at E8.5, but no longer expressed Gbx2 (Gbx2(GFP)−/ß-Gal+) at E12.5 were observed ventrally and dorsally along the full length of cord. The Gbx2 gene was turned off in these cells at some point between marking and E12.5 (H–J) ß-Gal antibody labeling of cells marked by GIFM at E8.5 versus antibody labeling to Pax2 on sagittal sections of an E12.5 embryo. Colocalization revealed that Gbx2(GFP) expressing cells marked at E8.5 gave rise to dorsal and ventral Pax2+ cells at all R-C levels; arrowheads show examples of co-localization. Sections shown here are from a medial plane, which precludes seeing dorsal Pax2+ cells because they are distributed in a “V-shaped” distribution (See Figure 1). (L–P) The Gbx2 lineage (ß-Gal+) marked at E8.5 by GIFM versus antibody labeling of Isl1/2+ cells. Colocalization revealed that Gbx2 expressing cells marked at E8.5 gave rise to dorsal and ventral Isl1/2+ cells at all R-C levels. (TIF) [file pone.0020940.s003.tif]

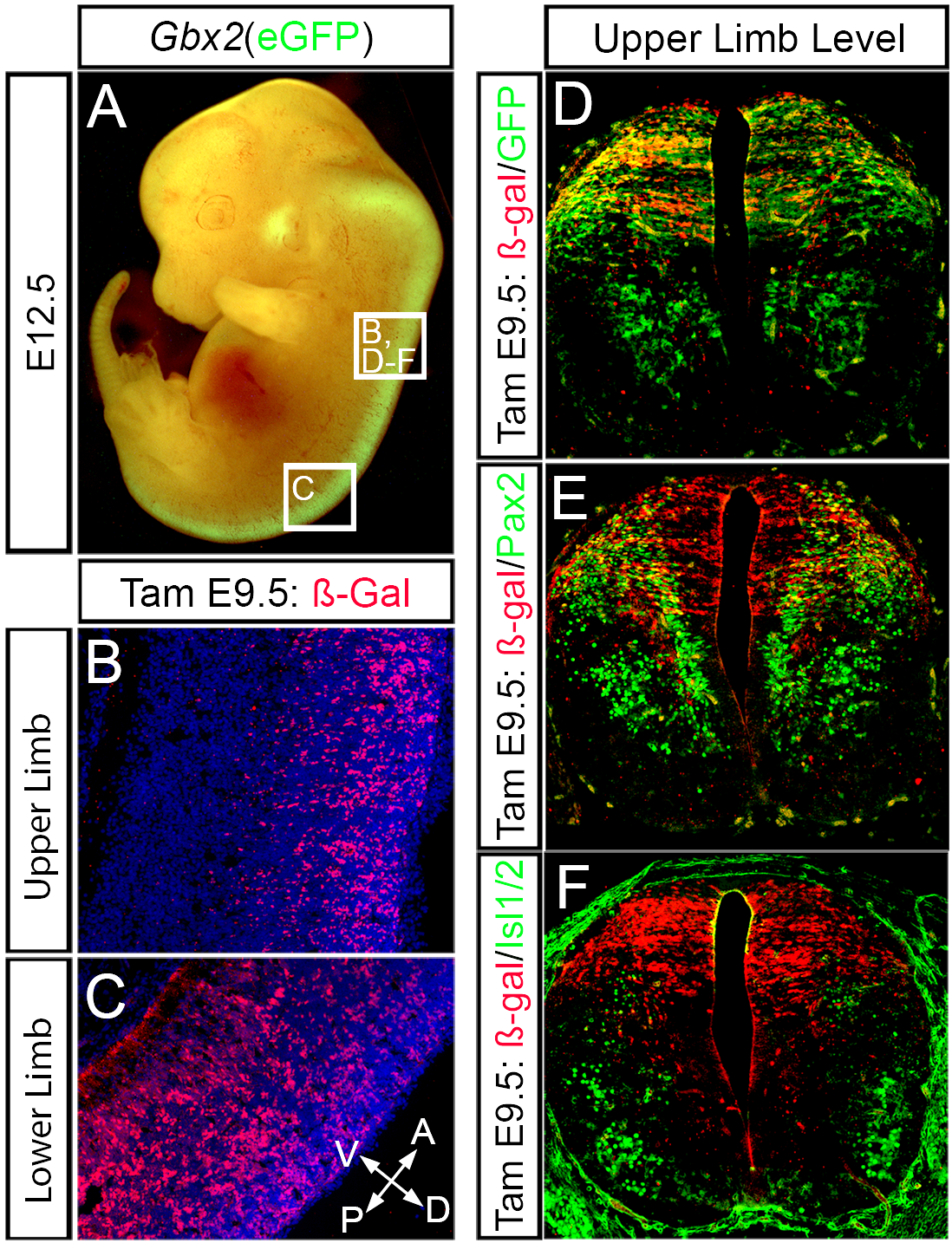

Supplement: Figure S4 — Gbx2 lineage marked at E9.5 makes varied contributions to the E12.5 spinal cord at different rostral caudal positions. (A) A lateral view of an E12.5 mouse embryo analyzed by wholemount Gbx2(GFP) fluorescence revealed Gbx2 expression. (B–E) GIFM by tamoxifen administration at E9.5 and analysis at E12.5 (B,C) Cells expressing Gbx2 at E9.5 and marked by GIFM that were detected by ß-Gal on sagittal sections gave rise to cells in dorsal cord at rostral levels (upper limb level) while at caudal levels (lower limb), the Gbx2 lineage gave rise to cells distributed across the full dorsal-ventral and medial-lateral extent of cord. (D) ß-Gal and GFP antibody labeling on transverse sections of cord at the upper limb level. Cells in the dorsal domain that were GFP+/ß-Gal+ continuously expressed Gbx2 from E9.5–E12.5. Cells in the ventral domain that were GFP+/ß-Gal− did not express Gbx2 at E9.5 but expressed Gbx2 at E12.5. (E) ß-Gal and Pax2 immunolabeling on transverse sections at the upper limb level of an E12.5 embryo revealed that Gbx2 expressing cells marked at E9.5 give rise to dorsal Pax2+ cells but only rarely to ventral Pax2+ cells. (F) ß-Gal and Isl1/2 immunolabeling on transverse sections showed marked cells were interspersed with dorsal Isl1/2+ cells but did not give rise to dorsal or ventral Isl1/2+ cells. (TIF) [file pone.0020940.s004.tif]

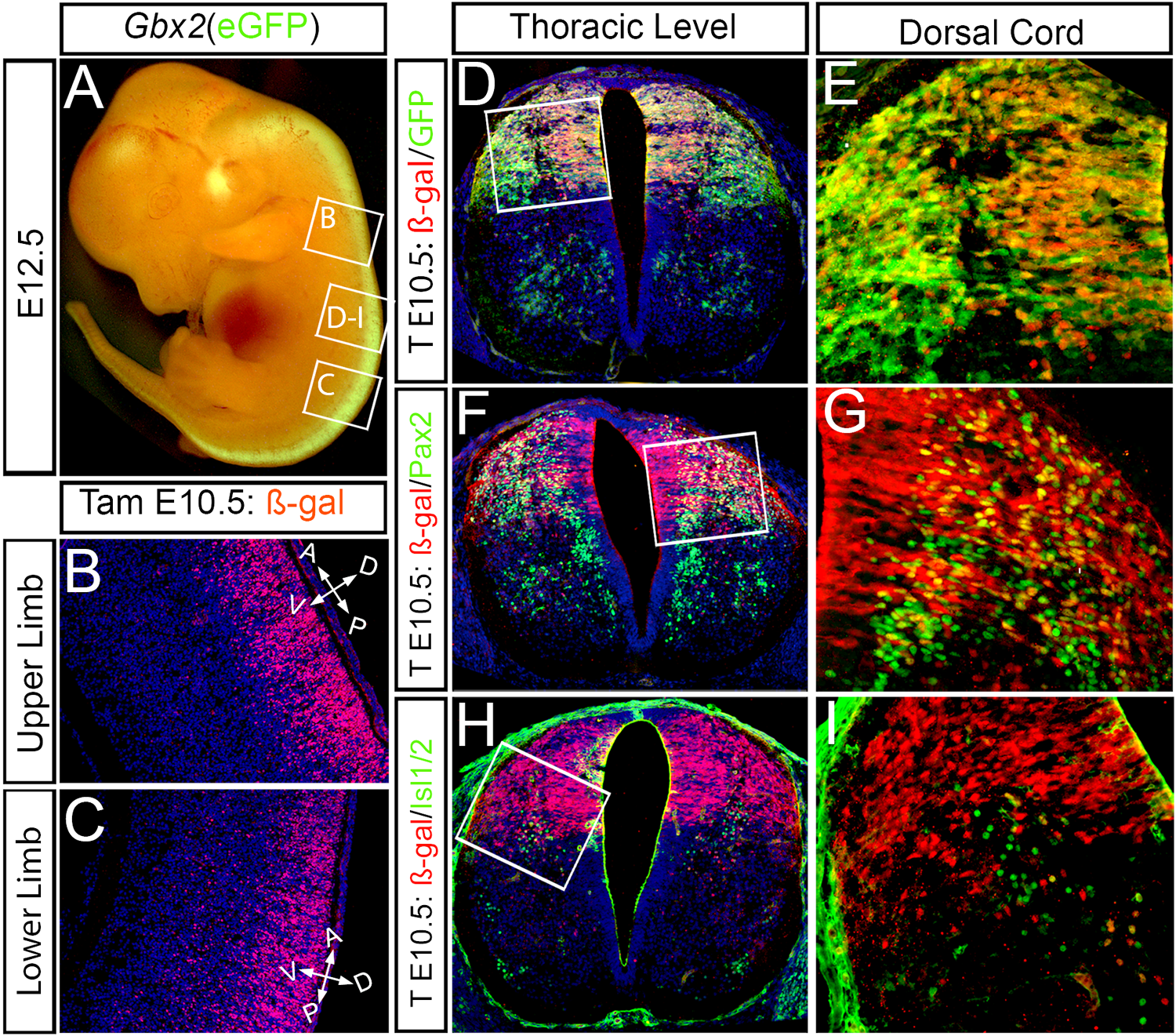

Supplement: Figure S5 — Gbx2 lineage marked at E10.5 contributes to dorsal spinal cord at E12.5. (A) A lateral view of an E12.5 mouse embryo analyzed by Gbx2(GFP) wholemount GFP fluorescence showed Gbx2 expression. (B,C) Cells that expressed Gbx2 at E10.5 were detected by ß-Gal immunolabeling on sagittal sections of rostral spinal cord. The Gbx2 lineage marked at E10.5 gave rise to a tightly restricted dorsal domain at the upper (B) and lower (C) limb level. (D,E) ß-Gal and GFP antibody labeling on thoracic level transverse sections. Most cells in the dorsal domain were Gbx2(GFP)+/ß-Gal+ and therefore had continuously expressed Gbx2 from E10.5–E12.5. There was no ß-Gal expression in ventral cord at the thoracic level. Cells in the ventral domain that were Gbx2(GFP)+/ß-Gal− did not express Gbx2 at E10.5, but did express Gbx2 at E12.5. (F,G) ß-Gal and Pax2 immunolabeling on thoracic level transverse sections revealed that Gbx2 expressing cells marked at E10.5 gave rise to dorsal Pax2+ cells but not ventral Pax2+ cells. (H,I) ß-Gal and Isl1/2 immunolabeling on thoracic level transverse sections were interspersed with dorsal Isl1/2+ cells but did not give rise to dorsal or ventral Isl1/2+ cells. (TIF) [file pone.0020940.s005.tif]

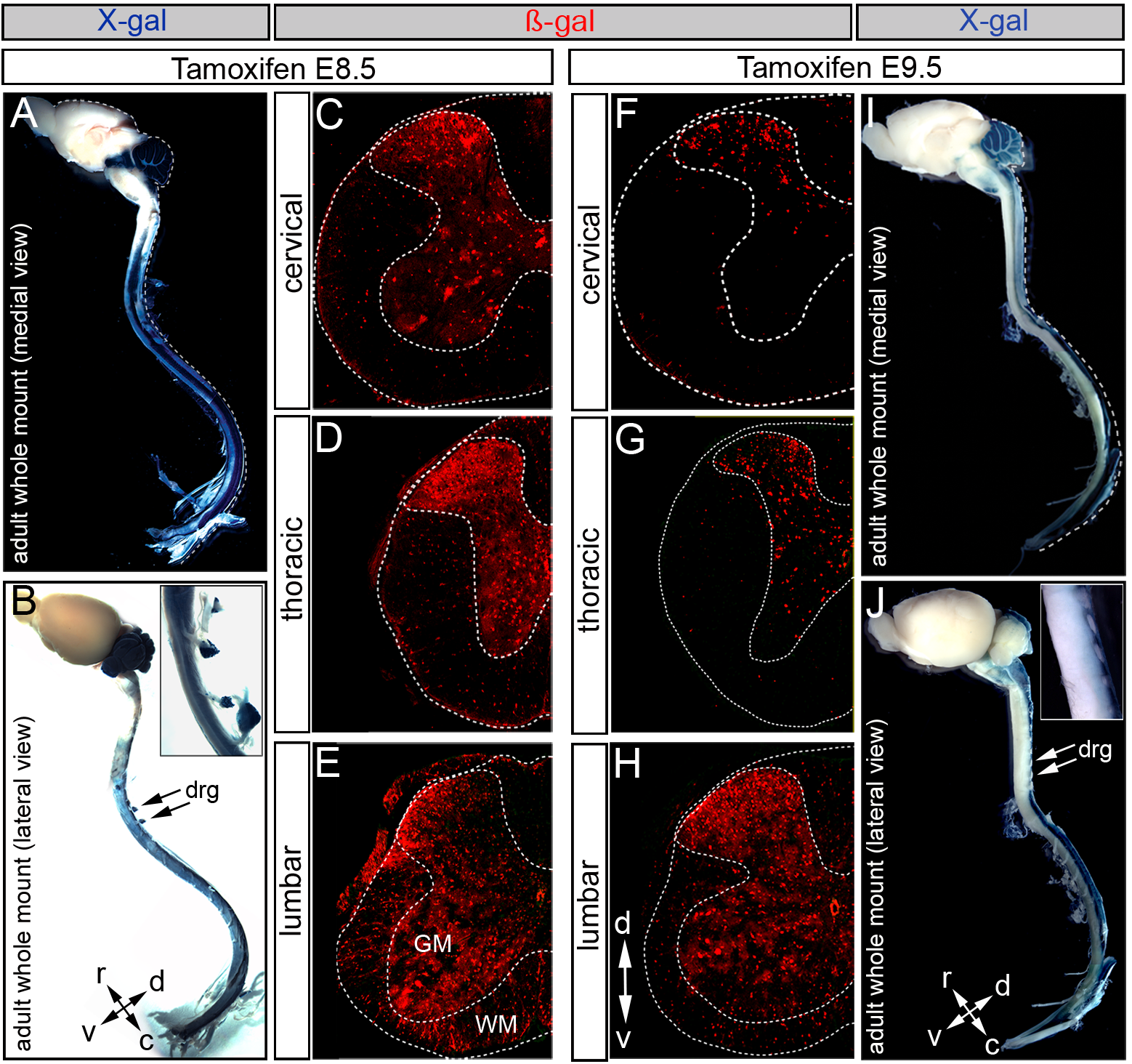

Supplement: Figure S6 — Distribution of Gbx2 lineage marked at E8.5 and E9.5 in adult spinal cord. (A–B) Sagittal view of whole mount brain and spinal cord processed for X-gal histochemistry show the Gbx2 lineage marked at E8.5. Panels A and B show medial and lateral CNS, respectively. (C–E) The Gbx2 lineage marked at E8.5 was detected on transverse hemi-sections of adult spinal cord at the cervical enlargement (upper limb level, C), thoracic flank level (D), and lumbar enlargement (lower limb level, E). The Gbx2 lineage (ß-Gal+) contributed to the full extent of the D-V spinal cord along the entire A-P axis including both grey and white matter (E). (F–H) The Gbx2 lineage (ß-gal+) marked at E9.5 at the upper limb (F), thoracic flank (G), and lower limb (H) levels. The Gbx2 lineage was restricted dorsally at the upper limb level but contributed to both dorsal and ventral domains at the lower limb levels although the contribution to white matter was less than when marked at E8.5. (I–J) Sagittal whole mount views of entire brain and spinal cord with X-gal histochemical staining showing cells marked at E9.5. Panels I and J show, respectively, medial and lateral CNS. Insets in panels B and J show DRG (arrows). (TIF) [file pone.0020940.s006.tif]
